# eIF2α phosphorylation-ATF4 axis-mediated transcriptional reprogramming mitigates mitochondrial impairment during ER stress

**DOI:** 10.1016/j.mocell.2024.100176

**Published:** 2025-01-03

**Authors:** Hien Thi Le, Jiyoung Yu, Hee Sung Ahn, Mi-Jeong Kim, In Gyeong Chae, Hyun-Nam Cho, Juhee Kim, Hye-Kyung Park, Hyuk Nam Kwon, Han-Jung Chae, Byoung Heon Kang, Jeong Kon Seo, Kyunggon Kim, Sung Hoon Back

**Affiliations:** 1 School of Biological Sciences, University of Ulsan, Ulsan 44610, Korea; 2 Asan Institute for Life Sciences, Asan Medical Center, Seoul 05505, Korea; 3 AMC Sciences, Asan Medical Center, Seoul 05505, Korea; 4 Department of Biological Sciences, School of Life Sciences, Ulsan National Institute of Science and Technology (UNIST), Ulsan 44919, Korea; 5 School of Pharmacy, Jeonbuk National University, Jeonju 54896, Korea; 6 Central Research Facilities (UCRF), Ulsan National Institute of Science and Technology (UNIST), Ulsan 44919, Korea; 7 Department of Digital Medicine, Asan Medical Center, University of Ulsan College of Medicine, Seoul 05505, Korea; 8 Basic-Clinical Convergence Research Center, School of Biological Sciences, University of Ulsan, Ulsan 44610, Korea

**Keywords:** Endoplasmic reticulum stress, Eukaryotic translation initiation factor 2α phosphorylation, Activating transcription factor 4, Nuclear factor erythroid 2-related factor 2, Mitochondrial homeostasis

## Abstract

Eukaryotic translation initiation factor 2α (eIF2α) phosphorylation, which regulates all 3 unfolded protein response pathways, helps maintain cellular homeostasis and overcome endoplasmic reticulum (ER) stress through transcriptional and translational reprogramming. However, transcriptional regulation of mitochondrial homeostasis by eIF2α phosphorylation during ER stress is not fully understood. Here, we report that the eIF2α phosphorylation-activating transcription factor 4 (ATF4) axis is required for the expression of multiple transcription factors, including nuclear factor erythroid 2-related factor 2 and its target genes responsible for mitochondrial homeostasis during ER stress. eIF2α phosphorylation-deficient (*A/A*) cells displayed dysregulated mitochondrial dynamics and mitochondrial DNA replication, decreased expression of oxidative phosphorylation complex proteins, and impaired mitochondrial functions during ER stress. ATF4 overexpression suppressed impairment of mitochondrial homeostasis in *A/A* cells during ER stress by promoting the expression of downstream transcription factors and their target genes. Our findings underscore the importance of the eIF2α phosphorylation-ATF4 axis for maintaining mitochondrial homeostasis through transcriptional reprogramming during ER stress.

## INTRODUCTION

Eukaryotic cells use the unfolded protein response (UPR), a set of signal transduction pathways, to manage endoplasmic reticulum (ER) stress, which is caused by disruptions in protein folding and glycosylation (Marciniak and Ron, 2006). The UPR begins with activation of the ER sensor IRE1, which triggers unusual splicing of *Xbp1* mRNA. This process produces the X-box-binding protein 1 (XBP1) transcription factor (TF), which promotes the transcription of genes that aid protein folding, secretion, and degradation during ER stress (Han and Kaufman, 2017, Walter and Ron, 2011). The UPR also involves 2 other signaling branches: activation of the transmembrane stress sensors activating transcription factor 6 (ATF6) and protein kinase R-like ER kinase (PERK). Under ER stress, proteolytic cleavage of ATF6 at the Golgi releases its N-terminal domain, allowing it to enter the nucleus and activate genes that enhance protein folding, degradation, secretion, and biogenesis of ER proteins (Han and Kaufman, 2017, Walter and Ron, 2011). PERK activation results in the phosphorylation of eIF2α, reducing general protein translation to decrease the protein load in the ER. Interestingly, eIF2α phosphorylation enhances the translation of specific genes, such as *Atf4*, which in turn activates genes related to redox balance, amino acid metabolism, autophagy, and protein synthesis (Dang et al., 2023, Walter and Ron, 2011, Young and Wek, 2016). Furthermore, eIF2α phosphorylation supports the expression of XBP1s and facilitates the activation of ATF6 by promoting its movement from the ER to the Golgi during stress (Dang et al., 2023, Teske et al., 2011). Hence, eIF2α phosphorylation, which influences all 3 UPR pathways, plays a crucial role in maintaining cellular homeostasis and overcoming stress through coordinated transcriptional and translational changes.

During ER stress, the UPR pathways communicate with or regulate cellular organelles other than the ER, such as mitochondria (Rainbolt et al., 2014, Zhang et al., 2023) and autophagosomes (Dang et al., 2023, Song et al., 2017), contributing to both cell survival and death. In addition, the ER and mitochondria form physical contact points through mitochondria-associated membranes (MAMs). MAMs allow a large amount of Ca^2+^ transfer from the ER to the mitochondria, stimulating mitochondrial bioenergetics or initiating apoptosis under ER stress conditions (Rainbolt et al., 2014, Zhang et al., 2023). However, several reports indicate that enhanced mitochondrial respiration promotes cell survival during the early phase of ER stress (Bravo et al., 2011, Hijazi et al., 2020, Knupp et al., 2019), proposing that an adaptive mitochondrial response to ER stress requires both IRE1 and calcium signaling via PERK and calcineurin (Bravo et al., 2011, Knupp et al., 2019). In addition, ER stress stimulates the formation of respiratory supercomplexes through the activation of the PERK-eIF2α phosphorylation-ATF4 axis to satisfy the energetic demands of ER stress responses (Balsa et al., 2019). The studies described above indicate that active mitochondria regulated by the UPR are required to adapt to or overcome ER stress. In this respect, mitochondrial dynamics such as fission and fusion may also be important mitochondrial responses to adapt to or overcome ER stress. Mitochondrial fission fragments damage mitochondria, which are then removed by mitophagy (Youle and van der Bliek, 2012), while mitochondrial fusion is a pro-survival mechanism that suppresses pathological mitochondrial fragmentation and enhances mitochondrial functions such as ATP production to protect cells under multiple stress conditions (Tondera et al., 2009, Wai and Langer, 2016). Mitochondrial fusion is mediated by large dynamin-related GTPase proteins, specifically optic atrophy 1 (OPA1), mitofusin 1 (MFN1), and mitofusin 2 (MFN2). Conversely, mitochondria fission requires the recruitment of dynamin-related protein 1 (DRP1) from the cytosol to the mitochondria. Dynamin-related protein 1 (DRP1) is a GTPase that associates with 4 DRP1 receptor proteins: mitochondrial fission protein 1 (FIS1), mitochondrial fission factor (MFF), and mitochondrial dynamics proteins of 49 kDa and 51 kDa (MID49 and MID51, respectively). These receptors are located on the mitochondrial outer membrane (Wai and Langer, 2016). It has been reported that peroxisome proliferator-activated receptor γ coactivator 1α (PGC1α) plays an important role in mitochondrial dynamics by modulating pivotal factors in these processes, including MFN1, MFN2, OPA1, DRP1, and FIS1 (Chen et al., 2022, Ding et al., 2018 Martin et al., 2014; Qian et al., 2024; Soriano et al., 2006; Sui et al., 2021). In addition, as a critical regulator of mitochondrial biogenesis, PGC1α upregulates nuclear respiratory factors 1 and 2α (NRF1 and 2α), which promote transcription of many mitochondrial genes, including those encoding subunits of mitochondrial respiratory chain/oxidative phosphorylation (OXPHOS) complexes (Bruni et al., 2010, Chen et al., 2009, Gleyzer et al., 2005, Scarpulla et al., 2012, Wu et al., 1999). NRF1 and NRF2α, together with PGC1α, stimulate the expression of mitochondrial transcription factor A (TFAM), which is a mitochondrial DNA (mtDNA)-binding protein essential for genome maintenance (Chen et al., 2022, Gleyzer et al., 2005, Kelly and Scarpulla, 2004, Vina et al., 2009). It has been reported that nuclear factor erythroid 2-related factor 2 (NFE2L2/Nrf2), a key TF for the antioxidant response (Gureev et al., 2019), regulates PGC1α at the transcriptional level (Baldelli et al., 2013, Deng et al., 2020, Gureev et al., 2019). It seems highly probable that Nrf2-mediated PGC1α expression occurs because it has been reported that PERK phosphorylates and activates Nrf2 during ER stress (Cullinan et al., 2003). Additionally, recent studies revealed that Nrf2 plays important roles in mitochondrial quality control by regulating the expression of NRF1 and TFAM (Gureev et al., 2019, Kasai et al., 2019, Piantadosi et al., 2008). Furthermore, PGC1α protein activity is regulated by AMP-activated protein kinase (AMPK)-mediated phosphorylation and AMPK-SIRT1-mediated deacetylation (Canto et al., 2009, Chen et al., 2022, Jager et al., 2007). In addition, it was reported that AMPK directly phosphorylates Nrf2 to tune the transactivation of selected target genes and that interplay occurs between AMPK and Nrf2 to readjust cellular homeostasis in various pathophysiological settings (Petsouki et al., 2022). Therefore, the AMPK and Nrf2-PGC1α-NRF1/2α-TFAM pathway is a critical contributor to maintaining the active mitochondrial quality control system, including mitochondrial dynamics and mtDNA replication. However, the UPR pathway-dependent regulation of this pathway is not well-studied.

In the present study, we revealed that the eIF2α phosphorylation-ATF4 axis is required for the expression of multiple TFs, including Nrf2 and its target genes responsible for mitochondrial dynamics and mtDNA replication during ER stress. Moreover, eIF2α phosphorylation-deficient (*A/A*) cells displayed impaired expression of OXPHOS complex proteins and dysregulated mitochondrial functions during ER stress. However, ATF4 overexpression (OE) significantly rescued or prevented all these mitochondrial defects in *A/A* cells under ER stress conditions. Our data demonstrate that the eIF2α phosphorylation-ATF4 axis maintains mitochondrial homeostasis by mediating transcriptional reprogramming under ER stress conditions.

## MATERIALS AND METHODS

### Cell Culture

All cell lines were incubated at 37°C with 5% CO_2_ in a humidified incubator. Wild-type (*S/S*) and eIF2α phosphorylation-deficient (*A/A*) MEFs (Dang et al., 2023) were cultured in Dulbecco’s modified Eagle’s medium (DMEM; Thermo Fisher Scientific, 11995065) supplemented with 10% dialyzed fetal bovine serum (FBS; Thermo Fisher Scientific, 26400044), 1% penicillin-streptomycin (WelGENE, LS202-02), and 1% MEM nonessential amino acids (NEAAs; Thermo Fisher Scientific, 11140-050). Immortalized hepatocytes (*S/S*^*Hep*^ and *A/A*^*Hep*^) (Dang et al., 2023) were cultured in Medium 199 (WelGENE, LM006-01) supplemented with 10% FBS (WelGENE, S001-01) and 1% penicillin-streptomycin. Wild-type (*Atf4*^*+/+*^) and ATF4-knockout (*Atf4*^*-/-*^) MEFs were cultured in DMEM supplemented with 10% FBS, 1% penicillin-streptomycin, 2% MEM amino acids (WelGENE, LS004-01), 1% MEM NEAAs (WelGENE, LS005-01), and 55 µM β-mercaptoethanol (Sigma-Aldrich, M3148) as previously described (Han et al., 2013).

### Recombinant Adenovirus Production

The empty control, ATF4/EGFP-expressing, and wild-type eIF2α-expressing adenoviral vectors [pShuttle-CMV, pAD-Track-ATF4, and pShuttle-CMV-eIF2α(WT)] were described previously (Dang et al., 2023). Recombinant adenoviruses expressing ATF4/EGFP or eIF2α(WT) using the AdEasy vector system (Agilent Technologies, 240009) were generated using a previously described procedure (Dang et al., 2023). The indicated MEFs were seeded into 100-mm culture dishes at a density of 7 × 10^5^ cells/dish or into 96-well culture dishes at a density of 6,000 cells/well, cultured for 12 to 16h, and infected with the indicated recombinant adenoviruses [*Ad-vector*, *Ad-ATF4/EGFP*, or *Ad-eIF2α(WT)*] for 24 h. The infected cells were treated with or without tunicamycin (Tm) for the indicated durations before undergoing various analyses, including quantitative PCR (qPCR), western blotting (WB), mitochondrial function assessments, oxidative stress marker assessments, and cell viability assays.

### Quantitative PCR

After the indicated treatments, total RNA was isolated from cells using QIAzol Lysis Reagent (QIAGEN, QI-79306). cDNA was synthesized using a High-Capacity cDNA RT Kit (Applied Biosystems, ABS-4368814). qPCR was performed using TOPreal SYBR Green qPCR High-ROX PreMIX (Enzynomics, RT501M) and a StepOnePlus Real-Time PCR System (Applied Biosystems). The specificity of each primer pair was confirmed by melting curve analysis. The levels of target mRNAs were normalized to that of *β-actin* (*β-act*) mRNA. The qPCR primer pairs used in this study are listed in Table S1.

### mtDNA Quantification

Genomic DNA was extracted using an AccuPrep Genomic DNA Extraction Kit (Bioneer, K-3032) according to the manufacturer’s instructions. Relative amounts of mtDNA and nuclear DNA were determined by qPCR. The ratio of mtDNA to nuclear DNA reflects the mtDNA content of a cell. qPCR was performed using a StepOnePlus Real-Time PCR System, 10 ng of total DNA, and Luna Universal qPCR Master Mix (New England BioLabs, M3003X). The nuclear gene *hexokinase 2* (*Hk2*) was used to compare 2 mtDNA-encoded genes (*mitochondrial-encoded cytochrome b* [*mt-cytb*] and *16s ribosomal RNA* [*mt-16s rRNA*]). The qPCR primer pairs used in this study are listed in Table S1. The mtDNA content was normalized by the nuclear DNA content.

### Western Blot Analysis

Cells were lysed in Nonidet P40 lysis buffer (1% IGEPAL CA-630 NP40, 50 mM Tris-HCl pH 7.5, 150 mM NaCl, 0.05% sodium dodecyl sulfate [SDS], 0.5 mM sodium orthovanadate, 100 mM NaF, 50 mM β-glycerophosphate, and Halt Protease Inhibitor Cocktail). Cell lysates were centrifuged at 13,000g for 15 min at 4°C and the supernatants were collected. For WB analysis of NFE2L2/Nrf2, cells were treated with Tm for the indicated durations and then with MG132 (20 µM) for 1 h before harvesting samples. Cells were directly lysed in SDS lysis buffer (1% SDS, 50 mM Tris-Cl pH 7.5, 150 mM NaCl, 0.5 mM sodium orthovanadate, 100 mM NaF, and 50 mM β-glycerophosphate) supplemented with Halt Protease Inhibitor Cocktail (Thermo Fisher Scientific, 1861279). The lysates were immediately heated for 15 min at 100°C. The homogenates were centrifuged at 13,000 g for 15 min at 4°C and the supernatants were collected. Protein concentrations were determined using a Pierce BCA Protein Assay Kit (Thermo Fisher Scientific, 23227). Cell lysates were subjected to WB analysis as described previously (Dang et al., 2023). Information regarding antibodies is provided in Table S2.

### Cell Imaging Using Confocal Microscopy

Cells were plated on collagen-coated 35-mm glass bottom confocal dishes (SPL Life Science, 101350) at a density of 2.5 × 10^5^ cells/dish. The next day, cells were treated with vehicle (Veh, DMSO) and either Tm (1 µg/ml) or thapsigargin (Tg, 500 nM) in phenol red-free DMEM (Gibco, 21063029) for the indicated durations. In ATF4 or eIF2α(WT) OE experiments, cells were infected with the indicated recombinant adenoviruses [*Ad-vector*, *Ad-ATF4/EGFP*, or *Ad-eIF2α(WT)*] for 24 h before Tm treatment. During the last 30 min of the chemical treatment, the cell culture medium was supplemented with MitoTracker Red (20 nM, Invitrogen, M7512) and tetramethylrhodamine methyl ester (TMRM; 30 nM, Thermo Fisher Scientific, T668) to stain cells. If nuclear staining was necessary, Hoechst 33258 (5 µg/ml, Sigma-Aldrich, 94403) was added together with the dye. Live-cell imaging was performed using a FV1200-OSR confocal microscope (Olympus). The staining intensity was measured using the mean fluorescence intensity (MFI) tool of FV10-ASW-4.2 software (Olympus).

### Mitochondrial Morphology Analysis

Images of MitoTracker Red-stained live cells on collagen-coated 35-mm glass bottom confocal dishes were recorded using a FV1200-OSR confocal microscope with a 60× oil objective (Olympus), a Hamamatsu ImagEM high-sensitivity camera (Hamamatsu Photonics), and FV10-ASW-4.2 software (Hamamatsu Photonics). For quantification, images of more than 50 cells per condition were collected, and a modified protocol of Lebeau et al. (2018) was used. Quantification was performed by blinding the images and then scoring cells based on the presence of primarily fragmented, tubular, or elongated mitochondria by 3 researchers. The scores of the 3 researchers were averaged and combined with the results of other independent experiments in the data presented in bar graphs.

### Data-Independent Acquisition Mass Spectrometry

To prepare samples for proteomics analysis, cell pellets were lysed using 5% SDS and 50 mM TEAB (pH 8.5) and boiled for 30 min at 80°C. After centrifugation at 13,000 rpm at 4°C, the supernatant was collected and the BCA assay was performed to measure the soluble protein concentration. Digestion was performed using the suspension trapping method (S-Trap, Profiti, C02-mini) according to the manufacturer’s instructions. After peptide desalting, the peptide mixture was dried and stored at −80°C for liquid chromatography-mass spectrometry analysis.

For peptide separation using mid-pH chromatography of a peptide library, 10 mM TEAB (pH 8.5) in high-performance liquid chromatography (HPLC) water was used as eluent A and 10 mM TEAB (pH 8.5) in 90% acetonitrile and 10% HPLC water was used as eluent B. The sample, which was 12 individually digested cell peptides (1 μg aliquot each) that had been pooled, was dissolved in 80 µl of eluent A and then injected into a 100 µl sample loop. Separation was performed on an XBridge Peptide BEH C-18 column (3 mm i.d. × 50-mm length; pore size, 130 Å; particle size, 2.5 µm; Waters Corporation) using a Shimadzu Prominence HPLC instrument at a flow rate of 0.25 ml/min for 51 min. A gradient of 5% B for 2 min, 5% to 40% B for 20 min, 40% to 44% B for 1.5 min, 44% to 60% B for 7 min, 60% to 5% B for 1.5 min, and 5% B for 19 min was applied. From 2 to 50 min, the eluents were collected in increments of 0.5 ml in a 96-well plate on an FRC-10A instrument (Shimadzu Prominence). The resulting 48 fractions were blended in a concatenate method to form 24 fractions. Each fraction was dried and stored at −80°C.

For data-dependent acquisition to build an in-house spectral library, 200 ng of tryptic peptides was attached to Evotips and separated using an Evosep One liquid chromatographer (Evostep) connected to a timsTOF Pro 2 instrument (Bruker). A C-18 column (15 cm × 75 µm; particle size, 1.9 µm) from PepSep (Bruker) and WHISPER100 20 SPD (60 min) were used in the Evosep One method. The mass spectrometer was operated in DDA-Parallel Accumulation-Serial Fragmentation (PASEF) mode. Ten PASEF MS/MS scans were triggered per cycle (1.17 s). DDA-PASEF parameters included the following: charge state, 1 to 5; mass/charge ratio (*m/z*) range, 100 to 1,700; mobility (1/K0) range, 0.60 to 1.6 V⋅s/cm^2^; lock duty cycle to 100%; capillary voltage, 1,600 V; dry gas, 3 l/min; dry temperature, 180°C; and accumulation and ramp time, 100 ms. The target intensity per individual PASEF precursor and intensity threshold were set to 20,000 and 2500, respectively, considering an active exclusion of 0.4-min elution. Mobility-dependent collision energy ramping was set to 59 and 20 eV at an inversed reduced mobility (1/K0) of 1.6 and 0.6 V·s/cm^2^, respectively. Collision energies were linearly interpolated between these 2 1/K0 values.

By using the FragPipe computational platform (v.19.1) with MSFragger (v.3.7) (Kong et al., 2017), Philosopher (v.4.8.1) (da Veiga Leprevost et al., 2020), and EasyPQP (v.0.1.37) components to build spectral libraries, peptides were identified from tandem mass spectra using the MSFragger search engine and raw (.d) files (24 fractionated dda-PASEF datasets) as input. *Mus musculus* protein sequence databases from UniProt (reviewed sequences only; downloaded on June 5, 2023) containing 17,155 protein sequences were used. Reversed protein sequences were appended to the original databases as decoys. Raw dda-PASEF files were analyzed with MSFragger using default closed search configurations, except the maximum variable modification was changed to 5. PSM validation with default closed search settings was applied (eg, 1% protein and 1% peptide-level false discovery rate [FDR]).

In data-independent acquisition for cell lysates, 200 ng of tryptic peptides was loaded onto Evotips and analyzed using an Evosep One liquid chromatographer (Evosep) connected to a timsTOF Pro 2 instrument (Bruker). The Evosep One method was WHISPER100 20 SPD, and the mass spectrometer was operated in 25 dia-PASEF scan mode, leading to a cycle time of 2.76 s. Data were acquired using 25-Da precursor window 100-ms scans (creating 25 windows). The parameters were as follows: *m/z* range, 341.1 to 1292.1, and mobility (1/K0) range, 0.61 to 1.37 V⋅s/cm^2^. The other mass spectrometer parameters were the same as those used in dda-PASEF mode.

In proteome database searches using DIA-NN, raw dia-PASEF files (12 files) were analyzed with DIA-NN (v.1.8.1) (Demichev et al., 2020) using the spectral library (8,608 protein groups and 115,051 precursors) with default settings, except the covered peptide length range was 7 to 30, the precursor charge range was 2 to 4, the precursor *m/z* range was 100 to 1,700, the fragment *m/z* range was 100 to 1,700, the maximum number of missed cleavages was 1, turning of protein inference to use the inference from the constructed library, cysteine carbamidomethylation was used as a fixed modification, N-terminal methionine excision, N-terminal acetylation, and methionine oxidation were used as variable modifications and quantification strategy to robust LC (high precision).

For statistical analysis of proteomics data, Perseus (https://maxquant.net/perseus/) was used to transform the relative protein abundance of each sample set from DIA-NN to log_2_ values and to perform normalization with width adjustment. Missing values were replaced using a normal distribution. An analysis of variance (ANOVA), the t-test, and cluster analysis were performed using the final normalized abundance values of 4 sample sets. Volcano plots were generated using EnhancedVolcano in R software (version 4.3.2). Gene ontology (GO) analysis was conducted using ShinyGO (ver 0.77, http://bioinformatics.sdstate.edu/go/) and differentially expressed proteins (DEPs) in each comparison.

### Mitochondrial Complex I Activity Assay

For the mitochondrial complex I activity assay, mitochondrial fractions were isolated using a Mitochondrial Isolation Kit (Abcam, 89874) according to the manufacturer’s instructions. Cells were plated in 150-mm culture dishes at a density of 2 × 10^6^ cells/dish, cultured for longer than 12 h, and treated with Veh (DMSO) and either Tm (1 µg/ml) or Tg (500 nM) for 24 h. In ATF4 or eIF2α(WT) OE experiments, cells were infected with the indicated recombinant adenoviruses (*Ad-vector*, *Ad-ATF4/EGFP*, or *Ad-eIF2α[WT*]) for 24 h before Tm or Tg treatment. Then, complex I activity in isolated mitochondria was measured using a Complex I Enzyme Activity Microplate Assay Kit (Abcam, ab109721) according to the manufacturer’s instructions. Briefly, the protein content was first measured using a Pierce BCA protein assay kit. Samples (30 µg/well) were added and the complex I target was immobilized on the microplate well, which had been precoated with complex I-specific antibodies. After incubating the sample for 3 h at room temperature, optical density at OD_450_ was measured at 1-min intervals for 30 min using a SpectraMax ID3 microplate reader. Activity was expressed as the change in absorbance per minute per amount of sample loaded into the well.

### Flow Cytometric Analyses of Cells Stained With JC-1

Cells were seeded into 6-well plates at a density of 2.5 × 10^5^ cells/well, cultured for longer than 12 h, treated with the specified chemicals for the indicated durations, harvested, and washed twice with cold PBS. Next, cells were stained with JC-1 (2 µM, Dojindo, MT09) for 45 min and PI (2 µg/ml, Sigma-Aldrich, P4864) for 15 min in the dark. Finally, cells were washed 3 times with PBS to remove unincorporated dyes and resuspended in 300 µl of PBS. Cells were then analyzed using a FACSCanto II flow cytometer (BD Biosciences) and FlowJo software (Ashland). The results are presented as dot plots and bar graphs.

### Measurement of the Cellular ATP Level

The intracellular ATP level was measured using a CellTiter-Glo 2.0 reagent kit (Promega, G9241) according to the manufacturer’s instructions. Cells were seeded on 96-well plates at a density of 8,000 cells/well, cultured for longer than 12 h, and treated with Veh (DMSO) and either Tm (1 µg/ml) or Tg (500 nM) for 24 h.

### Determination of the Redox State of Protein Disulfide Isomerase (PDI)

The redox state of PDI was determined using a modified protocol of Nagy et al. (2007). Cells were plated in 100-mm culture dishes at a density of 8 × 10^5^ cells/dish, cultured for longer than 12 h, and treated with Tm (1 µg/ml) for the indicated durations. In ATF4 OE experiments, cells were transfected with the indicated plasmids (*pCGN-Vec* or *pCGN-ATF4-IRES-EGFP*) for 24 h before Tm treatment. Protein lysates were prepared as described in the “Western Blot (WB) Analysis” section of the “MATERIALS and METHODS.” Lysates containing 40 µg of protein were diluted in 0.1 M Tris buffer (pH 7.2) to a volume of 40 µl. Samples were then precipitated with 5% (final concentration) trichloroacetic acid and centrifuged at 13,000 g for 5 min at 4°C. The pellets were washed twice with 70% acetone and resuspended in 40 µl of resolving buffer (0.1 M Tris, 250 mM SDS, and 6 M urea, pH 6.8). Thereafter, AMS (4-acetamido-4′-maleimidylstilbene-2,2′-disulfonic acid, disodium salt; Molecular Probe, A485) was added to samples at a final concentration of 20 mM and incubated for 15 min at 37°C. For reduced and oxidized control samples, lysates (40 µg) were incubated at 37°C for 15 min with 100 mM dithiothreitol (DTT; Thermo Fisher Scientific, R0861) and 25 mM diamide (Santa Cruz, sc-211289), respectively. Equal volumes of 2× sample buffer without reducing reagent were added to the samples, which were boiled for 5 min and loaded onto an 8% nonreducing SDS-PAGE gel, and WB analysis was performed.

### Statistical Analysis

All data are presented as mean ± standard error of the mean (SEM). All experiments were performed at least 3 times. Statistical analyses were performed using Student’s t-test, a 1-way ANOVA, or a 2-way ANOVA with GraphPad Prism 8.4.3 (GraphPad Software). Statistical significance is indicated in the figures (^*,#,&^*P* < .05, *^*,##,&&^*P* < .01, ^***,###,&&&^*P* < .001).

## RESULTS

### Mitochondrial Dynamics and mtDNA Replication Are Dysregulated in eIF2α Phosphorylation-Deficient (*A/A*) and ATF4-Deficient Cells During ER Stress

**Fig. 1:**
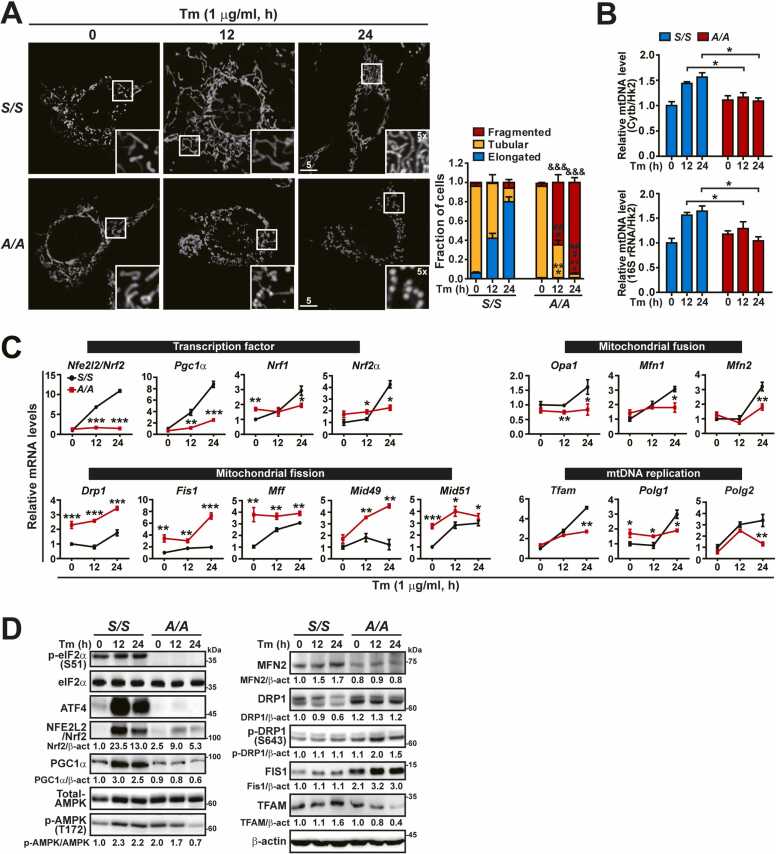
eIF2α phosphorylation is required for optimal expression of genes responsible for mitochondrial dynamics and mtDNA replication during ER stress. (A) Representative MitoTracker Red staining images of *S/S* and *A/A* MEFs. Cells were treated with Tm (1 µg/ml) for the indicated durations and stained with MitoTracker Red (white) for the last 30 min. The inset shows a 5× magnified image of the white boxed area. Scale bar: 5 µm. The graph shows the fractions of cells containing fragmented, tubular, and elongated mitochondria among *S/S* and *A/A* MEFs treated with Tm. Data are presented as mean ± SEM (at least 50 cells per condition). ***P < .001, *S/S* versus *A/A* at each time point of “Elongated”; ^###^*P* < .001, *S/S* versus *A/A* at each time point of “Tubular”; ^& & &^*P* < .001, *S/S* versus *A/A* at each time point of “Fragmented.” (B) qPCR analysis of mtDNA levels in *S/S* and *A/A* MEFs treated with Tm for the indicated durations. The mtDNA (*mt-cytb* and *mt-16s rRNA*) levels were normalized by the nuclear DNA (*Hk2*) level in each sample. Data are presented as mean ± SEM (n = 3). *P < .05, *S/S* versus *A/A* at each time point. (C) Quantitative RT-PCR analysis of mRNA levels of transcription factor-, and mitochondrial dynamics (fusion and fission)-, and mtDNA replication-related genes in *S/S* and *A/A* MEFs treated with Tm for the indicated durations. Data are presented as mean ± SEM (n = 3). *P < .05, **P < .01, and ***P < .001, *S/S* versus *A/A* at each time point. (D) WB analysis of mitochondrial dynamics- and mtDNA replication-related proteins in lysates of *S/S* and *A/A* MEFs treated with Tm for the indicated durations. Protein levels normalized by β-act or AMPK levels are shown below the panels.

During ER stress induced by treatment with the N-glycosylation inhibitor Tm (Fig. 1A) or the sarcoplasmic/endoplasmic reticulum Ca^2+^-ATPase inhibitor Tg (Fig. S1A), we compared mitochondrial morphology between wild-type (*S/S*) and eIF2α phosphorylation-deficient (*A/A*) MEFs. Mitochondria were visualized by MitoTracker Red staining (which labels the mitochondrial matrix) and mitochondrial morphology was quantified in blinded images by counting cells containing predominantly fragmented, tubular, or elongated mitochondria. As reported previously (Lebeau et al., 2018), mitochondria in *S/S* MEFs displayed ER stress-induced mitochondrial elongation/hyperfusion, but mitochondria in *A/A* MEFs were fragmented upon ER stress (Figs. 1A and S1A). Similar to *A/A* MEFs, eIF2α phosphorylation-deficient immortalized mouse embryonic hepatocytes (*A/A*^*Hep*^) displayed mitochondrial fragmentation in response to Tm and Tg treatments (Fig. S2A and B). Mitochondrial DNA (mtDNA) replication is correlated with mitochondrial fusion (Chen et al., 2010, Silva Ramos et al., 2019); therefore, mtDNA levels were measured. As expected, Tm and Tg treatments increased the level of mtDNA in *S/S* MEFs but not in *A/A* MEFs (Figs. 1B and S1B). Thus, eIF2α phosphorylation is required for mitochondrial dynamics and mtDNA replication under ER stress conditions. Phosphorylation of eIF2α is required for optimal expression of many UPR genes under ER stress conditions (Back et al., 2009, Dang et al., 2023, Majumder et al., 2012, Teske et al., 2011, Wek et al., 2006, Young and Wek, 2016). Among eIF2α phosphorylation-dependent genes, ATF4 is an important TF to maintain mitochondrial functions (Almeida et al., 2022, Kasai et al., 2019, Quiros et al., 2017). Therefore, we investigated whether ATF4 deficiency perturbs mitochondrial dynamics and mtDNA replication during ER stress. As expected, Tm and Tg treatments induced mitochondrial elongation in *Atf4*^*+/+*^ MEFs but mitochondrial fragmentation in *Atf4*^*-/-*^ MEFs (Fig. S3A-C). In addition, Tm treatment increased mtDNA levels in *Atf4*^*+/+*^ MEFs but not in *Atf4*^*-/-*^ MEFs (Fig. S3D). Thus, ATF4 is required for both mitochondrial dynamics and mtDNA replication during ER stress.

Expression of genes responsible for amino acid import and antioxidant responses is severely impaired in *Atf4*^*-/-*^ MEFs, and consequently, these cells are prone to amino acid depletion and are sensitive to oxidative stress (Dickhout et al., 2012, Harding et al., 2003, Lewerenz and Maher, 2009). Therefore, *Atf4*^*-/-*^ MEFs should be cultivated in a complete growth medium with extra supplements [nonessential amino acids (NEAAs) and the reducing agent β-mercaptoethanol]. Consequently, *Atf4*^*+/+*^ MEFs were cultured in the same medium. When NEAAs were removed from culture media, eIF2α phosphorylation gradually increased in both *Atf4*^*-/-*^ and *Atf4*^*+/+*^ MEFs (Fig. S3E). However, the expression of ATF4 and its downstream target proteins (CHOP and ASNS) was induced in *Atf4*^*+/+*^ MEFs but was not significantly changed at any time point in *Atf4*^*-/-*^ MEFs (Fig. S3E). These results indicate that the cells transiently experience a shortage of NEAAs, which activates the ATF4-dependent integrated stress response (Harding et al., 2003, Pakos-Zebrucka et al., 2016). Therefore, we checked whether nutrient stress induced by the removal of NEAAs changes mitochondrial morphology in MEFs. Removal of NEAAs induced mitochondrial elongation in *Atf4*^*+/+*^ MEFs but mitochondrial fragmentation in *Atf4*^*-/-*^ MEFs (Fig. S3F and G), suggesting that ATF4 is also required for regulation of mitochondrial dynamics under a stress condition induced by amino acid starvation. To reinforce the association between ATF4 and changes in mitochondrial morphology and mtDNA replication in Tm-treated cells, we restored ATF4 expression in *Atf4*^*-/-*^ MEFs and investigated mitochondrial morphology and the mtDNA content. As expected, recombinant adenovirus-mediated OE of ATF4/EGFP efficiently rescued the ER stress-induced defects of mitochondrial elongation and mtDNA replication (Fig. S3H-J). Our data indicate that eIF2α phosphorylation and ATF4 are required for the regulation of both mitochondrial dynamics and mtDNA replication in response to ER stress.

### eIF2α Phosphorylation Is Required for Optimal Expression of Multiple TFs and Their Target Genes Responsible for Mitochondrial Dynamics and mtDNA Replication During ER Stress

Multiple reports indicate that mitochondrial dynamics and mtDNA replication are regulated by transcriptional cascades of several TFs such as NFE2L2/Nrf2 (Baldelli et al., 2013, Deng et al., 2020, Gureev et al., 2019), PGC1α (Chen et al., 2022), NRF1 and 2α (Bruni et al., 2010, Gleyzer et al., 2005), and TFAM (Kang et al., 2018). Furthermore, PGC1α increases the expression of MFN1 and MFN2, as well as OPA1 (Martin et al., 2014, Soriano et al., 2006, Sui et al., 2021), while decreasing the expression of DRP1 and FIS1 (Chen et al., 2022, Ding et al., 2018 Sui et al., 2021), thereby mediating the balance between mitochondrial fusion and fission (Chen et al., 2022, Qian et al., 2024). Therefore, we investigated whether eIF2α phosphorylation deficiency affects the expression of these TFs during ER stress. Tm treatment gradually increased the mRNA levels of these TFs (*Nfe2l2*/*Nrf2*, *Pgc1α*, *Nrf1*, *Nrf2α*, and *Tfam*) in *S/S* MEFs, but significantly reduced them in *A/A* MEFs (Fig. 1C). In addition, expression of TF proteins (NFE2L2/Nrf2, PGC1α, and TFAM) was impaired or diminished in *A/A* MEFs during ER stress (Fig. 1D). Furthermore, defective expression of these genes during ER stress was reproduced in another eIF2α phosphorylation-deficient cell line (*A/A*^*Hep*^) (Fig. S2C and D). Recent reports suggest that ATF4 is responsible for the increased expression of *Nfe2l2/Nrf2* mRNA and its protein during ER stress (Kress et al., 2023, Sarcinelli et al., 2020). We consistently report that NFE2L2/Nrf2 expression is impaired in *Atf4*^*-/-*^ MEFs but is strongly induced in *Atf4*^*+/+*^ MEFs under various stress conditions, including ER stress, oxidative stress, and nutrient stress (Le et al., submitted to *Molecules and Cells*). In addition, based on Nrf2-dependent reporter assay experiments, we suggest that the expression of *Nfe2l2/Nrf2* mRNA and its protein is dysregulated due to the impairment of ATF4 expression in cells deficient in eIF2α phosphorylation (Le et al., submitted to *Molecules and Cells*).

Expression levels of *Pgc1α* mRNA and PGC1α protein were lower in both types of *A/A* cells than in both types of *S/S* cells (Figs. 1C, 1D, S2C, and S2D). This might be due to reduced NFE2L2/Nrf2 expression in *A/A* cells during ER stress because NFE2L2/Nrf2 regulates PGC1α at the transcriptional level (Baldelli et al., 2013, Deng et al., 2020, Gureev et al., 2019). Furthermore, PGC1α protein activity is regulated by AMPK-mediated phosphorylation and AMPK-SIRT1-mediated deacetylation (Canto et al., 2009, Chen et al., 2022, Jager et al., 2007). Therefore, we examined whether eIF2α phosphorylation deficiency influences AMPK phosphorylation upon Tm-induced ER stress. Tm treatment increased AMPK phosphorylation in *S/S* cells, but gradually decreased it in *A/A* cells, although the level of AMPK phosphorylation was higher in *A/A* cells than in *S/S* cells before Tm treatment (Figs. 1D and S2D). Therefore, eIF2α phosphorylation may play dual roles in AMPK-mediated regulation of PGC1α activity and NFE2L2/Nrf2-mediated *Pgc1α* mRNA expression. In addition, NFE2L2/Nrf2 directly influences *Nrf1* expression via binding to antioxidant response elements (AREs) in the *Nrf1* promoter (Piantadosi et al., 2008), and PGC1α increases *Nrf11* and *Nrf2α* mRNA expression levels and coactivates their transcriptional functions (Chen et al., 2022, Wu et al., 1999). As expected, the levels of *Nrf1* and *Nrf2α* mRNAs at 24 h were lower in *A/A* cells than in *S/S* cells during ER stress (Figs. 1C and S2C).

Next, we examined the expression of mitochondrial dynamics-related genes (Chen et al., 2022, Soriano et al., 2006, Wai and Langer, 2016, Wu et al., 1999), which can be regulated by PGC1α (Chen et al., 2022, Ding et al., 2018 Martin et al., 2014; Qian et al., 2024; Soriano et al., 2006; Sui et al., 2021) and unknown factor(s). Under ER stress conditions, the mRNA levels of mitochondrial fusion genes (*Opa1*, *Mfn1*, and *Mfn2*) were lower in *A/A* cells compared with *S/S* cells, while the mRNA levels of mitochondrial fission genes (*Drp1*, *Fis1*, *Mff*, *Mid49*, and *Mid51*) were significantly higher in *A/A* cells than in *S/S* cells (Figs. 1C and S2C). Consequently, the expression of MFN2 was lower, whereas the levels of DRP1 and FIS1 proteins were higher in *A/A* cells than in *S/S* cells during ER stress (Figs. 1D and S2D). Furthermore, phosphorylation of DRP1 at Ser-643 (Ser-637 in human DRP1), which can lead to mitochondrial fragmentation (Han et al., 2008, Wang et al., 2012), was increased in *A/A* cells but remained unchanged in *S/S* cells under ER stress conditions (Figs. 1D and S2D). These results indicate that mitochondria are fragmented in *A/A* cells during ER stress due to decreased expression of mitochondrial fusion genes and increased expression and activation of mitochondrial fission genes.

NRF1 and NRF2α, together with PGC1α, stimulate the expression of TFAM, which is a mtDNA-binding protein essential for genome maintenance (Chen et al., 2022, Gleyzer et al., 2005, Kelly and Scarpulla, 2004, Vina et al., 2009). Therefore, we examined expression levels of *Tfam* mRNA (Figs. 1C and S2C) and TFAM protein (Figs. 1D and S2D) in Tm-treated *S/S* and *A/A* cells. As expected, the *Tfam* mRNA level was lower in *A/A* cells than in *S/S* cells after Tm treatment for 24 h (Figs. 1C and S2C). Similarly, Tm treatment gradually decreased the TFAM protein level in *A/A* cells but increased it in *S/S* cells (Figs. 1D and S2D). In addition, mRNA expression of genes (*Polg1* and *Polg2*) encoding 2 subunits of mtDNA polymerase-γ, which is responsible for mtDNA replication, was decreased in Tm-treated *A/A* cells (Figs. 1C and S2C). NRF2α positively regulates *Polg2* mRNA expression (Bruni et al., 2010), and the TFAM level is associated with *Polg1* mRNA expression (Correia et al., 2011, Hance et al., 2005). Our data suggest that dysregulation of mtDNA levels is caused by impairment of the Nrf2-PGC1α-NRF1/2α-TFAM pathway and AMPK activation in *A/A* cells during ER stress.

Together, our data indicate that impairment of the ATF4-Nrf2-PGC1α-NRF1/2α transcriptional cascades, including AMPK activation, which affect expression and/or activation of MFN2 and DRP1 for mitochondrial dynamics as well as TFAM and PolG1/2 for mtDNA replication, dysregulates mitochondrial dynamics and mtDNA replication in eIF2α phosphorylation-deficient cells during ER stress.

### ATF4 Is Required for Expression of Genes Responsible for Mitochondrial Dynamics and mtDNA Replication in *A/A* Cells During ER Stress

**Fig. 2:**
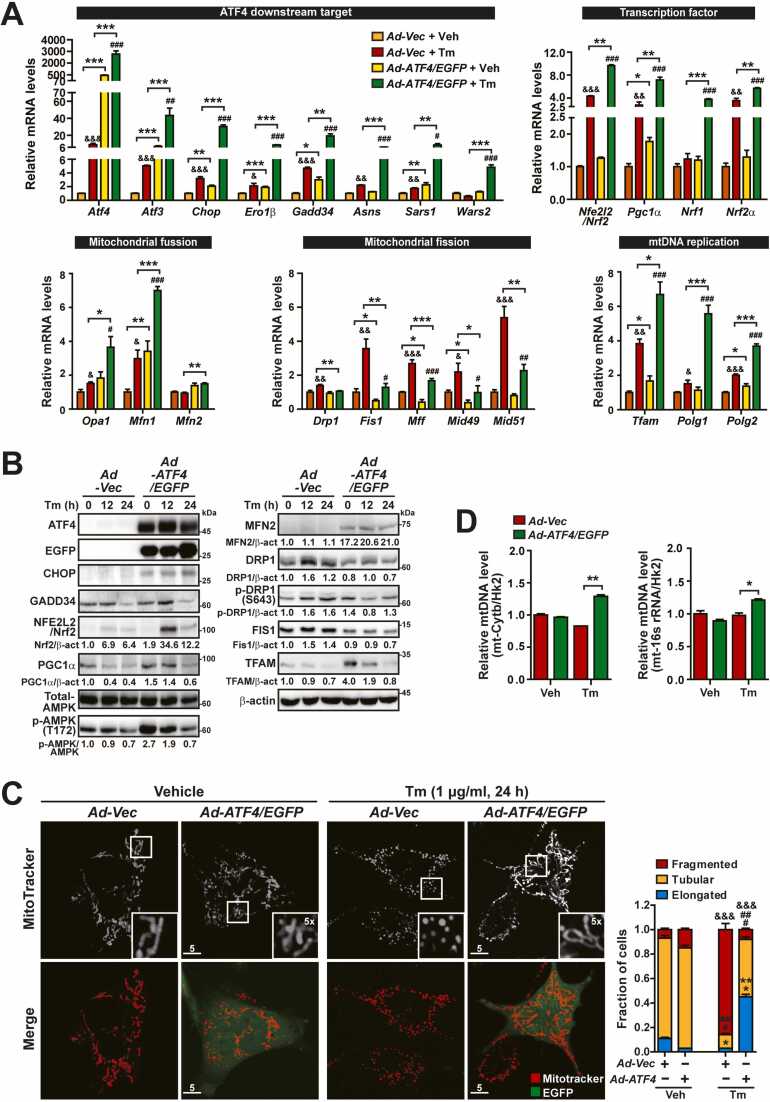
ATF4 OE rescues impairment of mitochondrial dynamics and mtDNA replication in *A/A* cells during ER stress. (A) Quantitative RT-PCR analysis of mRNA levels of *Atf4* and its downstream target-, transcription factor-, and mitochondrial dynamics (fusion and fission)- and mtDNA replication-related genes in Vector (Vec)- or ATF4/EGFP-overexpressing *A/A* MEFs treated with Veh (DMSO) or Tm for 24 h. Data are presented as mean ± SEM (n = 3). *P < .05, **P < .01, and ***P < .001, *Vec* versus *ATF4/EGFP*; ^&^*P* < .05, ^&&^*P* < .01, and ^&&&^*P* < .001, Veh versus Tm in *Vec;*^#^*P* < .05, ^##^*P* < .01, and ^###^*P* < .001, Veh versus Tm in *ATF4/EGFP*. (B) WB analysis of ATF4 and its downstream proteins and mitochondrial dynamics- and mtDNA replication-related proteins in lysates of Vec- or ATF4-/EGFP-overexpressing *A/A* MEFs treated with Tm for the indicated durations. Protein levels normalized by β-act or AMPK levels are shown below the panels. (C) Representative MitoTracker Red staining images of Vec- or ATF4-/EGFP-overexpressing *A/A* MEFs. *A/A* MEFs infected with Vec- or ATF4-/EGFP-expressing adenoviruses for 24 h were treated with Veh or Tm for 24 h and stained with MitoTracker Red (white) for the last 30 min. Expression of ATF4 is indicated by the green fluorescence of EGFP. The inset shows a 5× magnified image of the white boxed area. Scale bar: 5 µm. The graph shows the fractions of cells containing fragmented, tubular, and elongated mitochondria among Vec- or ATF4-/EGFP-overexpressing *A/A* MEFs treated with Veh or Tm. Data are presented as mean ± SEM (at least 50 cells per condition). *P < .05 and ***P < .001, *Vec* versus *ATF4/EGFP* in “Elongated”; ^###^*P* < .001, *Vec* versus *ATF4/EGFP* in “Tubular”; ^&&&^*P* < .001, *Vec* versus *ATF4/EGFP* in “Fragmented.” (D) qPCR analysis of mtDNA levels in Vec- or ATF4-/EGFP-overexpressing *A/A* MEFs treated with Tm for 24 h. The mtDNA (*mt-cytb* and *mt-16s rRNA*) levels were normalized by the nuclear DNA (*Hk2*) level in each sample. Data are presented as mean ± SEM (n = 3). *P < .05 and **P < .01, *Vec* versus *ATF4/EGFP*.

ATF4 OE rescued the dysregulation of mitochondrial dynamics and mtDNA replication in *ATF4*^*-/-*^ cells during ER stress (Fig. S3I and J). Therefore, we investigated whether it prevents dysregulated expression of genes related to mitochondrial dynamics and mtDNA replication in eIF2α phosphorylation-deficient cells during ER stress. To this end, *A/A* MEFs were infected with *Ad-Vector* (*Ad-Vec*) or *Ad-ATF4/EGFP* and then treated with or without Tm. qPCR and WB analyses confirmed that ATF4 was overexpressed and harbored transcriptional activities, as judged by increased mRNA and/or protein expression of ATF4 downstream genes (*Atf3*, *Chop*, *Ero1β*, *Gadd34*, *Asns*, *Sars1*, and *Wars2*) (Han et al., 2013, Harding et al., 2003, Wortel et al., 2017) (Fig. 2A upper-left panel and Fig. 2B). Expression analysis of TFs responsible for mitochondrial dynamics and mtDNA replication demonstrated that the mRNA and/or protein levels of *Nfe2l2*/*Nrf2, Pgc1α*, *Nrf1*, *Nrf2α*, and *Tfam* were significantly higher in *Ad-ATF4/EGFP-*infected cells than in *Ad-Vector-*infected cells under ER stress conditions, although there were discrepancies between the mRNA and protein levels of some genes (Fig. 2A upper-right panel and Fig. 2B). However, the Nrf2 level upregulated by ATF4 OE decreased after 24 h (Fig. 2B). This event was possibly due to natural degradation via the ubiquitin-proteasome pathway (Kobayashi et al., 2004) because it also occurred in wild-type (*S/S*) cells under Tm-treated conditions (Fig. 1D). In addition, similar to PGC1α and TFAM expression levels, AMPK phosphorylation in ATF4-overexpressing *A/A* MEFs was increased without Tm treatment and gradually decreased with Tm treatment (Fig. 2B), indicating that an eIF2α phosphorylation-deficient *A/A* cell may have another unknown defect that cannot be prevented by transient ATF4 OE under ER stress conditions.

ATF4 OE increased the mRNA levels of all mitochondrial fusion genes (*Opa1*, *Mfn1*, and *Mfn2*) while reducing the mRNA levels of all mitochondrial fission genes (*Drp1*, *Fis1*, *Mff*, *Mid49*, and *Mid51*) during ER stress (Fig. 2A). As a result, ATF4 OE increased the MFN2 protein level but decreased the levels of the DRP1, phosphorylated DRP1, and FIS1 protein in *A/A* cells during ER stress (Fig. 2B). Thus, the restoration of ATF4 expression rescued the dysregulated expression of genes related to mitochondrial dynamics, which is necessary for mitochondrial elongation, as well as TFAM, which is required for mtDNA replication. This restoration occurred due to the impairment of the ATF4-Nrf2-PGC1α-NRF1/2α transcriptional cascades, including AMPK activation, in *A/A* cells during ER stress.

Finally, we investigated whether activation of genes related to mitochondrial dynamics and mtDNA replication restores these processes in *A/A* MEFs during ER stress. As expected, defects of mitochondrial elongation and mtDNA replication were efficiently rescued in *Ad-ATF4/EGFP-*infected *A/A* cells but not in *Ad-Vector-*infected *A/A* cells under ER stress conditions (Fig. 2C and D). Our data indicate that ATF4 is required for expression of downstream TFs and their target genes responsible for mitochondrial dynamics and mtDNA replication in eIF2α phosphorylation-deficient cells during ER stress.

### eIF2α Phosphorylation Is Required for Optimal Expression of OXPHOS Complex Subunits During ER Stress

**Fig. 3:**
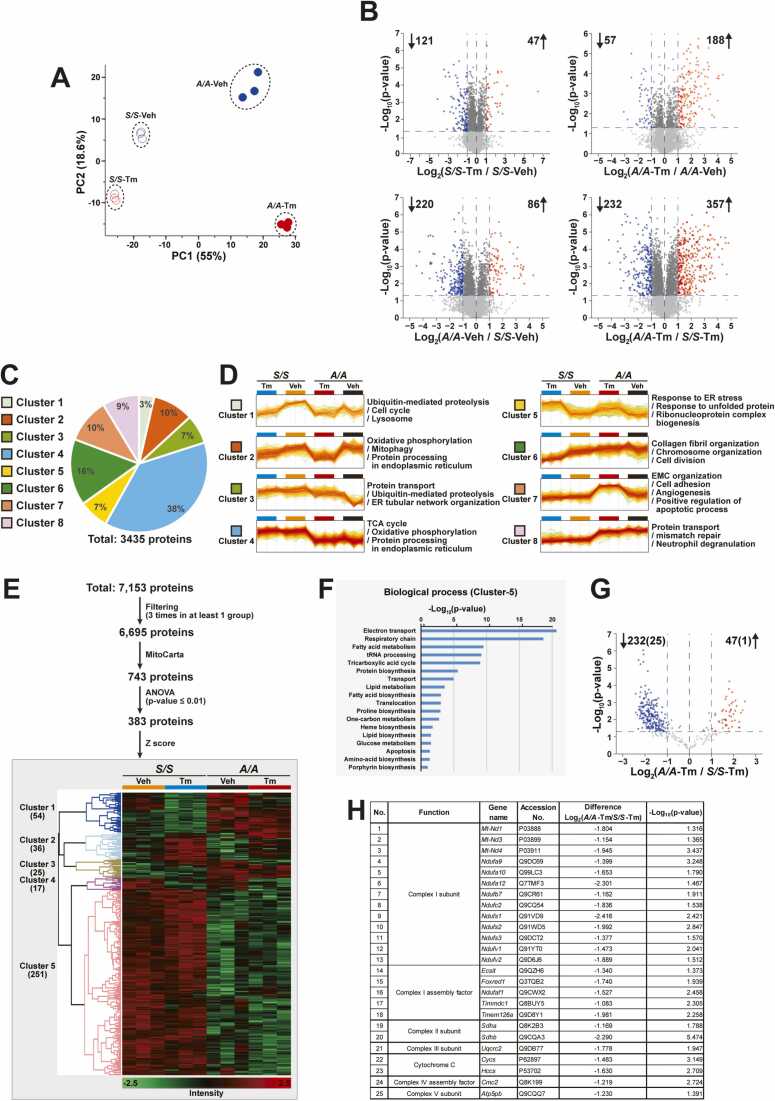
Expression of OXPHOS complex proteins is decreased in *A/A* cells during ER stress. (A) Principal component analysis of quantified proteins in 4 experimental groups (*S/S*-Veh, *A/A*-Veh, *S/S*-Tm, and *A/A*-Tm) treated with Veh or Tm for 24 h. (B) Volcano plots illustrating significant DEPs between the experimental groups. Each dot indicates a protein. Blue dots denote significantly downregulated proteins (log_2_ fold change < −1 and adjusted *P*-value < .05). Red dots denote significantly upregulated proteins (log_2_ fold change > 1 and adjusted *P*-value < .05). (C) Pie chart illustrating the number of clusters in HCL analysis. All 3,435 DEPs were grouped into 8 clusters with percentages. Hierarchical clusters of significant proteins determined by an ANOVA of the 4 experimental groups were analyzed using Perseus software (ver. 2.0.10.0). (D) Expression profiles of the 8 hierarchical clusters, which were significantly different from each other, and significantly enriched biological process GO terms in clusters. (E) HCL analysis of the 383 most abundant mitochondrial DEPs between the 4 experimental groups (ANOVA and *t*-test, permutation-based FDR < 0.01). The number of proteins in each cluster is indicated in parentheses. This figure was generated using Perseus software. Rows represent proteins and columns represent samples. High and low expression is shown in red and green, respectively. (F) Mitochondrial DEPs in cluster 5 of panel E underwent GO enrichment analysis (GO biological process) using the DAVID website. Minus log_10_ p-values of enriched proteins were plotted using Excel. (G) Volcano plot illustrating significant DEPs between the *S/S*-Tm and *A/A*-Tm groups. Blue and red dots denote significantly downregulated (232 downregulated proteins and 25 downregulated OXPHOS complex proteins) and upregulated (47 upregulated proteins and 1 upregulated OXPHOS complex protein) mitochondrial proteins, respectively. (H) List of OXPHOS complex subunits that were significantly downregulated in the *A/A*-Tm group compared with the *S/S*-Tm group.

To obtain global insight into the effect of ER stress on mitochondria in eIF2α phosphorylation-deficient cells at the proteome level, we performed comprehensive proteomics analysis of triplicate samples from 4 groups (*S/S*-Veh, *A/A*-Veh, *S/S*-Tm [24 h], and *A/A*-Tm [24 h]) using data-independent acquisition mass spectrometry. Two-dimensional principal component analysis plots using quantitative protein data showed good clustering of biological replicates and clear separation of the 4 groups, indicating that the quantitative protein profiles of these groups have different characteristics (Fig. 3A). Volcano plots of DEPs in the 4 groups are shown in Figure 3B. Using criteria of a log_2_ fold change > 1 and a −log_10_ (*P*-value) > 1.3, there were 168, 245, 306, and 589 significant DEPs in *S/S*-Veh versus *S/S*-Tm, *A/A*-Veh versus *A/A*-Tm, *S/S*-Veh versus *A/A*-Veh, and *S/S*-Tm versus *A/A*-Tm, respectively (Fig. 3B). These results indicated that significant differences in the levels of protein expression are induced by eIF2α phosphorylation deficiency as well as ER stress. However, there were more significant DEPs upon eIF2α phosphorylation deficiency than upon ER stress (306 and 589 vs 168 and 245), revealing that eIF2α phosphorylation is extremely important for the regulation of proper expression of multiple proteins during ER stress. To investigate the biological processes of mitochondria adversely affected by eIF2α phosphorylation deficiency before and/or after Tm treatment, we performed hierarchical clustering (HCL) and GO enrichment analyses. HCL analysis revealed 8 separate clusters (Fig. 3C) comprising upregulated and downregulated proteins compared with *S/S*-Veh (Fig. 3D). Clusters 2 (10%) and 4 (38%) were enriched in mitochondrial proteins involved in OXPHOS, which were downregulated by Tm treatment and eIF2α phosphorylation deficiency, respectively (Fig. 3D). To further analyze mitochondrial DEPs, MitoCarta-annotated data (743 proteins) were imported into Perseus and filtered by an ANOVA of the 4 groups with a permutation-based FDR ≤ 0.01, and 383 mitochondrial proteins were selected. By performing HCL analysis using Z-scores, we identified 5 clusters (Fig. 3E). Cluster 5 contained 251 mitochondrial proteins (65.5% of total proteins) whose expression was low in both *A/A*-Veh and *A/A*-Tm, indicating that the expression of these proteins is dependent on eIF2α phosphorylation. GO term enrichment (GO biological process) indicated that the most enriched biological processes in cluster 5 were electron transport (24 proteins) and the respiratory chain (19 proteins) (Fig. 3F), suggesting that eIF2α phosphorylation plays important roles in these processes. A volcano plot of mitochondrial DEPs in the *S/S*-Tm and *A/A*-Tm groups identified 279 mitochondrial proteins (232 downregulated and 47 upregulated) (Fig. 3G). Among them, 25 and 1 proteins were downregulated and upregulated OXPHOS complex proteins, respectively. Among the 25 downregulated OXPHOS complex proteins, the most affected proteins were complex I-related proteins, including complex I subunits (13 proteins) and complex I assembly factors (5 proteins) (Fig. 3H), indicating that ∼30% of total complex I subunits (44 proteins) (Rath et al., 2021) were downregulated in *A/A* MEFs during ER stress. In addition, half (*Sdha* and *Sdhb*) of all complex II subunits (4 proteins) were downregulated. eIF2α phosphorylation deficiency affected the expression of cytochrome C (*Cycs*) and its synthesizing enzyme (holocytochrome c-type synthase, *Hccs*). Our proteomics analysis indicated that eIF2α phosphorylation is required for the expression of diverse mitochondrial proteins, including OXPHOS complex proteins, which are important to maintain mitochondrial functions during ER stress.

### The eIF2α Phosphorylation-ATF4 Pathway Is Required to Maintain Mitochondrial Functions During ER Stress

**Fig. 4:**
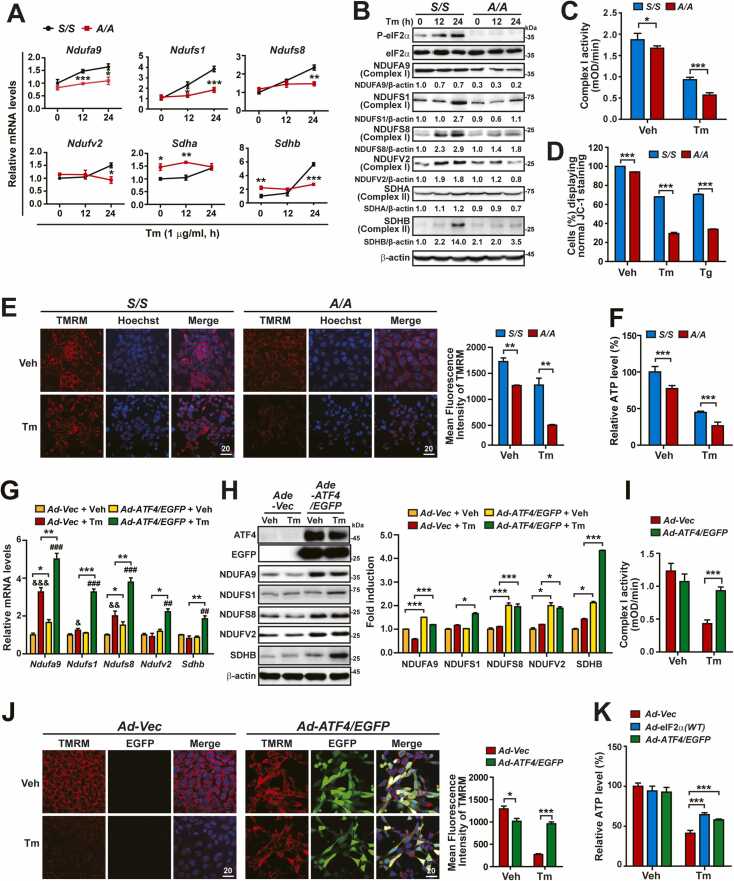
ATF4 OE suppresses mitochondrial dysfunction in *A/A* cells during ER stress. (A) Quantitative RT-PCR analysis of mRNA levels of OXPHOS genes in *S/S* and *A/A* MEFs treated with Tm for the indicated durations. Data are presented as mean ± SEM (n = 3). *P < .05, **P < .01, and ***P < .001, *S/S* versus *A/A* at each time point. (B) WB analysis of OXPHOS complex subunits in lysates of *S/S* and *A/A* MEFs treated with Tm for the indicated durations. Protein levels normalized by β-act levels are shown below the panels. (C) Mitochondrial complex I activity in *S/S* and *A/A* MEFs treated with Veh or Tm for 24 h. Data are presented as mean ± SEM (n = 3). *P < .05 and ***P < .001, *S/S* versus *A/A*. (D) The percentage of cells displaying normal JC-1 staining among *S/S* and *A/A* MEFs. Cells were treated with Veh, Tm, or Tg for 24 h and stained with JC-1 (red and green) for the last 30 min. The graph depicts a decrease in the number of red and green double-positive cells from the flow cytometric results in Figure S1D. Data are presented as mean ± SEM (n = 3). ***P < .001, *S/S* versus *A/A*. (E) Representative TMRM staining images of *S/S* and *A/A* MEFs. Cells were treated with Veh or Tm for 24 h and stained with TMRM (red) and Hoechst 33258 (blue) for the last 30 min. Scale bar: 20 µm. The graph depicts the quantification of the MFI of TMRM. Data are presented as mean ± SEM (n = 3, 15 random fields per condition). **P < .01, *S/S* versus *A/A*. (F) Measurement of total ATP levels in *S/S* and *A/A* MEFs treated with Veh or Tm for 24 h. Data are presented as mean ± SEM (n = 3). ***P < .001, *S/S* versus *A/A*. (G) Quantitative RT-PCR analysis of mRNA expression of OXPHOS complex I genes in Vec- or ATF4-/EGFP-overexpressing *A/A* MEFs treated with Veh or Tm for 24 h. Data are presented as mean ± SEM (n = 3). *P < .05, **P < .01, and ***P < .001, *Vec* versus *ATF4/EGFP*; ^&^*P* < .05, ^&&^*P* < .01, and ^&&&^*P* < .001, Veh versus Tm in *Vec*; ^##^*P* < .01 and ^###^*P* < .001, Veh versus Tm in *ATF4/EGFP*. (H) WB analysis of OXPHOS complex I subunits in lysates of Vec- or ATF4-/EGFP-overexpressing *A/A* MEFs treated with Veh or Tm for 24 h. The graph depicts the protein level normalized by the β-act level. Data are presented as mean ± SEM (n = 3). *P < .05 and ***P < .001, *Vec* versus *ATF4/EGFP*. (I) Mitochondrial complex I activity in Vec- or ATF4-/EGFP-overexpressing *A/A* MEFs treated with Tm for 24 h. Data are presented as mean ± SEM (n = 3). *P < .05, *Vec* versus *ATF4/EGFP*. (J) Representative TMRM staining images of Vec- or ATF4-/EGFP-overexpressing *A/A* MEFs. *A/A* MEFs infected with Vec- or ATF4-/EGFP-expressing adenoviruses for 24 h were treated with Veh or Tm for 24 h and stained with TMRM (red) and Hoechst 33258 (blue) for the last 30 min. Expression of ATF4 is indicated by the green fluorescence of EGFP. The graph depicts the quantification of the MFI of TMRM. Data are presented as mean ± SEM (n = 3, 15 random fields per condition). *P < .05 and ***P < .001, *Vec* versus *ATF4/EGFP*. (K) Measurement of total ATP levels in Vec-, eIF2α-WT-, or ATF4-/EGFP-overexpressing *A/A* MEFs treated with Veh or Tm for 24 h. Data are presented as mean ± SEM (n = 3). ***P < .001, *Vec* versus *eIF2α-WT* or *ATF4/EGFP*.

Proteomics analyses have indicated that eIF2α phosphorylation is required for proper expression of OXPHOS complex proteins during ER stress. To investigate whether eIF2α phosphorylation deficiency affects the expression of these proteins during ER stress, we performed qPCR and WB analyses of *S/S* and *A/A* cells treated with and without Tm. Consistent with the proteomics analyses (Fig. 3H), these analyses revealed that the mRNA and/or protein levels of complex I (*Ndufa9*, *Ndufs1*, *Ndufs8*, and *Ndufv2*) and complex II (*Sdha* and *Sdhb*) components, except for *Sdha* mRNA, were lower in *A/A* MEFs than in *S/S* MEFs under ER stress conditions (Fig. 4A and B). Similarly, the mRNA and/or protein expression levels of complex I (*Ndufa9*, *Ndufs1*, *Ndufs8*, and *Ndufv2*) and complex II (*Sdhb*) components, except for NDUFV2 protein, were lower in *A/A*^*Hep*^ cells than in *S/S*^*Hep*^ cells under ER stress conditions (Fig. S2E and F). These data indicate that eIF2α phosphorylation is required for the proper expression of multiple OXPHOS proteins under ER stress conditions. However, the protein level of NDUFA9 was lower in *A/A* MEFs than in *S/S* MEFs, and the protein levels of NDUFA9, NDUFS1, NDUFS8, and NDUFV2 were lower in *A/A*^*Hep*^ cells than in *S/S*^*Hep*^ cells without ER stress (Figs. 4B and S2F), suggesting that eIF2α phosphorylation regulates expression of some OXPHOS proteins even under normal conditions. Consistent with the qPCR and WB analyses, complex I activity was lower in *A/A* MEFs than in *S/S* MEFs during ER stress, although it decreased in both *S/S* and *A/A* MEFs following Tm and Tg treatments (Figs. 4C and S1C). Next, to assess whether ER stress causes mitochondrial dysfunction in *A/A* cells with dysregulated expression of OXPHOS complex proteins, the mitochondrial membrane potential (MMP) was determined using the ratiometric MMP indicator JC-1 (Figs. 4D and S1D) and the potentiometric MMP indicator TMRM (Figs. 4E, S1E, and S2G). The decrease in red and green double-positive cells, which are normal cells in JC-1-stained samples, was used to represent the change of the MMP in flow cytometric analysis (Fig. S1D). Upon Tm and Tg treatments, the number of red and green double-positive cells decreased much more among *A/A* MEFs than among *S/S* MEFs, although it decreased among both *S/S* and *A/A* cells (Fig. 4D). In addition, the fluorescence intensity of TMRM staining was lower in *A/A* cells than in *S/S* cells, although it was decreased in both *S/S* and *A/A* cells upon Tm and Tg treatments (Figs. 4E, S1E, and S2G). These data indicate that eIF2α phosphorylation is required to suppress the ER stress-mediated decrease of the MMP. Finally, total cellular ATP levels were measured to indirectly assess mitochondrial functions. As expected, the ATP level was much lower in *A/A* cells than in *S/S* cells during ER stress (Figs. 4F and S1F). However, unlike the differences in complex 1 activity and MMP between *S/S* and *A/A* cells before and after Tm and Tg treatments, the difference in the ATP level was not exacerbated by ER stress. Together, these results suggest that eIF2α phosphorylation is required for optimal expression of OXPHOS complex proteins and maintenance of mitochondrial functions during ER stress.

ATF4 OE rescued dysregulated expression of genes related to mitochondrial dynamics and mtDNA replication at the transcriptional level and thereby restored related phenotypes in eIF2α phosphorylation-deficient cells during ER stress. Therefore, we postulated that it may promote mRNA and protein expression of OXPHOS complexes and thereby enhance mitochondrial functions in *A/A* cells during ER stress. To investigate this, *A/A* MEFs were infected with *Ad-Vector* or *Ad-ATF4/EGFP* and then treated with Tm. Expression analysis of complex I (*Ndufa9*, *Ndufs1*, *Ndufs8*, and *Ndufv2*) and complex II (*Sdhb*) components demonstrated that their protein levels, except for NDUFS1, were significantly higher in *Ad-ATF4/EGFP-*infected cells than in *Ad-Vector-*infected cells regardless of Tm treatment, although their mRNA levels, except for *Ndufa9*, were only increased by Tm treatment in ATF4-overexpressing cells (Fig. 4G and H). In addition, ATF4 OE increased the complex 1 activity in Tm-treated *A/A* MEFs (Fig. 4I). Furthermore, ATF4-overexpressing *A/A* MEFs displayed significant increases in the intensity of TMRM staining after Tm treatment, although ATF4 OE slightly reduced the MMP in *A/A* MEFs before Tm treatment (Fig. 4J). These results indicate that ATF4 prevents a decrease of the MMP in *A/A* cells under ER stress conditions. Finally, we examined whether ATF4 or wild-type eIF2α OE increases the ATP level in *A/A* MEFs under Tm-treated conditions. As expected, ATF4 OE increased the ATP level similar to wild-type eIF2α OE in *A/A* MEFs during ER stress, although the ATP level was not further increased in *A/A* MEFs overexpressing ATF4 or eIF2α before Tm treatment (Fig. 4K). Our results indicate that the eIF2α phosphorylation-ATF4 pathway is required for proper expression of OXPHOS complex proteins and thereby suppresses impairment of mitochondrial functions in *A/A* cells during ER stress.

In Fig. 2, Fig. 4, we observed that ATF4 OE alleviated most mitochondrial dysfunctions (such as dysregulation of mitochondrial dynamics, mtDNA replication, and MMP) in *A/A* MEFs during ER stress. The functions of the ER and mitochondria are independent but interrelated because they form physical contact points through MAMs to regulate the physiological functions of both organelles through Ca^2+^, lipid, and metabolite exchange (Zhang et al., 2023). Therefore, recovery of mitochondrial functions might affect ER functions or vice versa in *A/A* cells during ER stress. In addition, as a TF, ATF4 controls the activation of ATF6α and expression of several genes (such as *Ero1β* in Fig. 1E) related to ER functions (Han et al., 2013, Harding et al., 2003, Teske et al., 2011). Therefore, we investigated whether ATF4 OE rescues dysregulated ER functions, including the aberrant ER structure (Back et al., 2009, Dang et al., 2023) in *A/A* cells during ER stress. Microscopic observation of an ER-targeted red fluorescent protein (DsRed-ER) revealed that the net-like ER network disappeared in *A/A* MEFs but not in *S/S* MEFs during ER stress (Fig. S4A). Furthermore, we analyzed the redox state of the ER chaperone PDI, which transfers oxidative equivalents to newly synthesized ER proteins and is a central protein in oxidative protein folding catalyzed in the ER of eukaryotic cells (Frand and Kaiser, 1999). During ER stress, levels of PDI and oxidized PDI were decreased in *A/A* MEFs but increased in *S/S* MEFs (Fig. S4B and C). However, ATF4 OE did not recover the net-like ER network or increase the levels of PDI and oxidized PDI in Tm-treated *A/A* MEFs (Fig. S4D-F). These results indicate that ATF4 is a critical TF to maintain the function of mitochondria, not the ER, although it contributes to the expression of some ER-related proteins (such as ERO1) during ER stress (Han et al., 2013, Harding et al., 2003, Teske et al., 2011).

## DISCUSSION

**Fig. 5:**
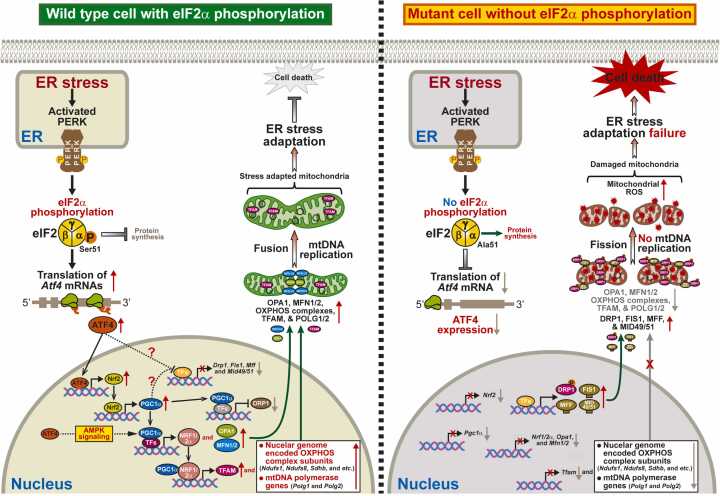
Molecular mechanisms by which the eIF2α phosphorylation-ATF4 axis maintains mitochondrial homeostasis through transcriptional reprogramming during ER stress.

The present study demonstrated that eIF2α phosphorylation plays an essential role in preservation of mitochondrial homeostasis by mediating transcriptional reprogramming during ER stress. We revealed that ATF4 overexpression, which was significantly downregulated in *A/A* cells during ER stress, ameliorated the impairment of mitochondrial homeostasis (mitochondrial dynamics, mtDNA replication, MMP, and OXPHOS) by promoting expression of several TFs and their target genes. Collectively, our results reveal how eIF2α phosphorylation induces transcriptional reprogramming via its downstream TF ATF4 to overcome or adapt to ER stress (Fig. 5).

As reported previously by the Wiseman group (Lebeau et al., 2018, Perea et al., 2023), we found that ER stress promotes mitochondrial elongation. They proposed that PERK activation and eIF2α phosphorylation increase both cellular phosphatidic acids (PAs) and ATP-dependent YME1L (inner membrane protease)-dependent degradation of the intramitochondrial PA transporter PRELID1 in response to ER stress (Perea et al., 2023). These events lead to the accumulation of PA on the outer mitochondrial membrane, where it promotes mitochondrial elongation by inhibiting mitochondrial fission through mechanisms such as direct inhibition of DRP1 (Adachi et al., 2016). However, mitochondrial dynamics are regulated by diverse mechanisms, including Ca^2+^/calmodulin-dependent protein kinase Iα (CaMKIα)- or Rho-associated coiled-coil-containing protein kinase 1-dependent phosphorylation of DRP1 at Ser-643 (human Ser-637), which leads to mitochondrial fragmentation (Han et al., 2008, Wang et al., 2012), and an increase of the total DRP1 or MFN2 protein level, which promotes mitochondrial fragmentation or elongation, respectively (Wu et al., 2011b). In addition, our data indicated that the ER stress inducer (Tm) upregulated the mRNA levels of mitochondrial fusion genes (*Opa1*, *Mfn1*, and *Mfn2*) and MFN2 protein levels in wild-type (*S/S*) cells (Figs. 1C, 1D, S2C, and S2D). However, during ER stress, *A/A* cells exhibited reduced mRNA levels of all mitochondrial fusion genes (*Opa1*, *Mfn1*, and *Mfn2*) and MFN2 protein levels (Figs. 1C, 1D, S2C, and S2D), along with increased mRNA levels of all mitochondrial fission genes (*Drp1*, *Fis1*, *Mff*, *Mid49*, and *Mid51*), elevated levels of DRP1 and FIS1 protein, and increased DRP1 phosphorylation (Figs. 1C, 1D, S2C, and S2D), leading to mitochondrial fission (Fig. 1A). By contrast, ATF4 OE in Tm-treated *A/A* cells reversed the expression patterns of all these mitochondrial fusion and fission genes (Fig. 2A and B), thereby inducing mitochondrial elongation (Fig. 2C). Furthermore, *Atf4*^*-/-*^ cells exhibited ER stress-dependent mitochondrial fission, not mitochondrial elongation, although eIF2α phosphorylation was increased (Fig. S3A-C). Therefore, ATF4 OE in Tm-treated *Atf4*^*-/-*^ cells recovered mitochondrial elongation during ER stress (Fig. S3H and I). However, there is a discrepancy in ER stress-dependent mitochondrial elongation in ATF4-deficient (*Atf4*^*-/-*^) MEFs between our results and those of the Wiseman group (Lebeau et al., 2018). In response to ER stress, we observed mitochondrial fragmentation in *Atf4*^*-/-*^ MEFs, but the Wiseman group did not. This discrepancy may be due to differences in treatment times (longer than 24 h vs 6 h) or MEF sources. We suggest that ER stress-dependent mitochondrial elongation requires eIF2α phosphorylation-ATF4 pathway-dependent transcriptional activity to modulate fission and fusion mediators, although upstream PERK-eIF2α phosphorylation-dependent translation regulation may be required to remodel mitochondrial phospholipids, as recently reported by the Wiseman group (Perea et al., 2023).

We suggest that the impairment of ATF4-dependent transcriptional cascades, including AMPK activation, is responsible for the dysregulated expression of genes related to mitochondrial dynamics and mtDNA replication in *A/A* cells during ER stress. First, it is possible that activated AMPK contributes to mitochondrial elongation and mtDNA replication via activation of Nrf2 (Petsouki et al., 2022) and/or PGC1α (Canto et al., 2009, Chen et al., 2022, Jager et al., 2007) during ER stress. Multiple studies, including our results (Figs. 1D and S2D), have reported that AMPK is phosphorylated in response to ER stress (Gong et al., 2023, Liu et al., 2016, Tamas et al., 2006). Liu et al. (2016) suggested that IRE1α participates in the activation of AMPK in response to Tm treatment. However, eIF2α phosphorylation deficiency does not change IRE1α activation during ER stress (Dang et al., 2023). In addition, ATF4 OE increased AMPK phosphorylation in *A/A* cells, although the level was gradually decreased by Tm treatment (Fig. 2B). Therefore, we propose that the eIF2α phosphorylation-ATF4 pathway partly contributes to AMPK activation in our experimental conditions. However, we did not determine how the eIF2α phosphorylation-ATF4 pathway activates AMPK and how this activation contributes to mitochondrial elongation and mtDNA replication through the activation of Nrf2 and/or PGC1α during ER stress. Further studies are required to answer many questions about the detailed molecular mechanisms underlying ATF4-mediated AMPK phosphorylation and its functional roles in mitochondrial elongation and mtDNA replication during ER stress.

Second, we suggest that *Nrf2* is the primary target gene of ATF4 and may play a role in mitochondrial elongation and mtDNA replication because Nrf2 regulates the expression of *Pgc1α*, which is a pivotal TF for these processes (Chen et al., 2022, Ding et al., 2018, Soriano et al., 2006) at the transcriptional level (Baldelli et al., 2013, Deng et al., 2020, Gureev et al., 2019). Additionally, ATF4 OE restored the expression levels of *Pgc1α* mRNA and its protein in *A/A* cells during ER stress (Fig. 2A and B). Consistent with these results, 3 recent reports (Kress et al., 2023, Sarcinelli et al., 2020), including our own (Le et al., submitted to *Molecules and Cells*), suggest that ATF4 directly controls the expression of *Nrf2* mRNA. Therefore, Nrf2 may be the second missing TF in *A/A* cells during ER stress. Wu et al. (2011a) and ourselves (Misra et al., 2013) reported that several ER stress inducers (Tm, Tg, DTT, and 2-deoxy-D-glucose) increase the expression of *Pgc1α* mRNA and its protein in primary myotubes, differentiated white fat cells (3T3-L1 cells), and a hepatocyte cell line (AML12), although specific ER stress inducers exhibit cell-type-specific regulation of *Pgc1α* mRNA expression (Wu et al., 2011a). Although we suggested that the eIF2α phosphorylation-ATF4-Nrf2 pathway is responsible for PGC1α expression, it is also possible that PGC1α expression can be regulated by transcription factor EB (TFEB) at the transcriptional level because PGC1α is a direct target of TFEB (Kim et al., 2018, Settembre et al., 2013), which is activated (Martina et al., 2016) through the eIF2α phosphorylation-ATF6α pathway during ER stress (Dang et al., 2023). However, the eIF2α phosphorylation-ATF4 pathway is required for the activation of ATF6α during ER stress (Dang et al., 2023, Teske et al., 2011). Nrf2 and/or TFEB might be required for PGC1α expression during ER stress. Therefore, further studies are needed to investigate which pathway(s) (ATF4-Nrf2 and/or ATF6α-TFEB) is required for PGC1α expression during ER stress.

Finally, given that ATF4 OE rescued the altered expression of mitochondrial fusion genes (*Opa1*, *Mfn1*, and *Mfn2*) and mitochondrial fission genes (*Drp1* and *Fis1*), thereby inducing mitochondrial elongation in *A/A* cells during ER stress (Fig. 2A and B), we propose that PGC1α expression may contribute to the decreased mRNA levels of mitochondrial fusion genes (*Opa1*, *Mfn1*, and *Mfn2*) and MFN2 protein levels, as well as to the increased mRNA levels of mitochondrial fission genes (*Drp1* and *Fis1*) and their corresponding DRP1 and FIS1 protein levels (Figs. 1C, 1D, S2C, and S2D). These results are congruent with prior studies on the roles of PGC1α in the modulation of OPA1, MFN1, MFN2, DRP1, and FIS1 expression (Chen et al., 2022, Ding et al., 2018 Martin et al., 2014; Qian et al., 2024; Soriano et al., 2006; Sui et al., 2021). Further studies are needed to investigate how PGC1α modulates the expression of these mitochondrial fusion and fission genes in *A/A* cells during ER stress. Moreover, there is currently no plausible explanation for why the expression of *Mff*, *Mid49*, and *Mid51* genes is increased, or how ATF4 OE rescues their altered expression in *A/A* cells during ER stress (Figs. 2A and B). Therefore, it is worthwhile to explore whether PGC1α can also modulate the expression of *Mff*, *Mid49,* and *Mid51* genes in *A/A* cells during ER stress. In addition, we showed that ATF4 OE suppressed DRP1 phosphorylation at Ser-643 in *A/A* cells during ER stress (Figs. 1D and 2B). Therefore, as previously reported (Han et al., 2008, Wang et al., 2012), DRP1 phosphorylated at Ser-643 may be involved in the increase of mitochondrial fission in *A/A* cells during ER stress. However, we have not identified which gene(s) is responsible for DRP1 phosphorylation or investigated how ATF4 suppresses this phosphorylation in *A/A* cells during ER stress. These issues require further investigation. Nevertheless, our findings indicate that an eIF2α phosphorylation-dependent transcriptional cascade (such as ATF4-Nrf2-PGC1α) with AMPK activation is required to modulate the expression and/or activity of mitochondrial fission and fusion mediators (such as OPA1, MFN1, MFN2, DRP1, and FIS1) for ER stress-induced mitochondrial elongation (Fig. 5), although mitochondrial elongation may also require eIF2α phosphorylation-mediated translational regulation to remodel the mitochondrial membrane (Perea et al., 2023).

Mitochondrial fusion is correlated with mtDNA replication (Chen et al., 2010, Silva Ramos et al., 2019), which is regulated by TFAM (Chen et al., 2022, Vina et al., 2009). In addition, the TFAM level is associated with *Polg1* mRNA expression (Correia et al., 2011, Hance et al., 2005), and NRF2α positively regulates *Polg2* mRNA expression (Bruni et al., 2010). Therefore, the reduced mtDNA levels in *A/A* cells during ER stress can be explained by reduced TFAM expression. TFAM may be downregulated due to reduced levels of Nrf2 and the PGC1α/NRF1/NRF2α complex because Nrf2 regulates expression of TFAM and NRF1 (Gureev et al., 2019, Kasai et al., 2019, Piantadosi et al., 2008), and NRF1 and NRF2α together with PGC1α stimulate expression of TFAM (Chen et al., 2022, Gleyzer et al., 2005, Kelly and Scarpulla, 2004, Vina et al., 2009) (Fig. 5). ATF4 OE promoted the Nrf2-PGC1α-NRF1/2α-TFAM transcriptional cascade and thereby induced mtDNA replication in *A/A* cells during ER stress. Collectively, our results revealed how phosphorylated eIF2α uses downstream TFs to regulate mtDNA levels during ER stress.

Proteomics analysis revealed that eIF2α phosphorylation was required for optimal expression of OXPHOS complex proteins (especially complex I and II subunits and cytochrome C) during ER stress (Fig. 3H), although eIF2α phosphorylation deficiency also affected the expression of many other mitochondrial proteins (Fig. 3E-H). Consistent with the results of proteomics analysis, qPCR and WB analyses confirmed that mRNA and/or protein levels of most observed complex I and II subunits were low in *A/A* cells during ER stress (Figs. 4A, 4B, S2E, and S2F). In addition, as expected, ATF4 OE enhanced the mRNA and/or protein levels of complex I and II subunits and thereby suppressed mitochondrial dysfunctions (decreases of complex I activity, MMP, and ATP levels) in *A/A* cells during ER stress (Fig. 4G-K). These results indicate that eIF2α phosphorylation/ATF4-mediated transcriptional reprogramming is required for proper expression of OXPHOS complex proteins and possibly other mitochondrial proteins and thereby maintains mitochondrial functions during ER stress. However, ATF4 OE alone could not restore mitochondrial dysfunctions (decreases of complex I activity, MMP, and ATP levels) in *A/A* cells under normal conditions (Fig. 4I-K vs Figs. 4C-F, S2C-F, and S2G). We propose 3 potential scenarios to explain why ATF4 OE could not restore mitochondrial dysfunctions under normal conditions. First, ATF4 expression is induced when eIF2α phosphorylation occurs and possibly other conditions are met during ER stress (Young and Wek, 2016). Therefore, ATF4 protein was barely or weakly detected under normal conditions (Figs. 1D, S2D, S3C, and S3E), suggesting that ATF4 does not play a critical role in mitochondrial functions without ER stress. Consequently, it is understandable that ATF4 OE alone cannot restore mitochondrial dysfunctions (such as decreases in complex I activity, MMP, and ATP levels) under normal conditions but can recover these functions during ER stress in *A/A* cells. In other words, the results suggest that mitochondrial dysfunctions in eIF2α phosphorylation-deficient *A/A* cells under normal conditions are due to the absence of an unknown factor, aside from ATF4. These issues require further investigation. Second, ATF4 OE enhanced the expression level of only *Ndufa9* mRNA among the observed OXPHOS genes before Tm treatment (Fig. 4G). In addition, it partly restored the protein levels of complex I and II subunits because NDUFS1 protein was not upregulated before Tm treatment (Fig. 4H). Furthermore, expression of ATF4-dependent UPR genes, as well as *Nrf2* and its downstream target genes, was not changed or only weakly induced by ATF4 OE in *A/A* MEFs before Tm treatment. However, Tm treatment significantly enhanced their expression (Fig. 2, Fig. 4). Therefore, we think that the effect of ATF4 OE alone is not strong enough to restore mitochondrial dysfunctions in *A/A* cells under normal conditions. Therefore, ATF4 might require functional activation through post-translational modifications (PTMs; ubiquitination, SUMOylation, acetylation, and phosphorylation) (Neill and Masson, 2023). However, these PTMs may not occur under normal conditions because they might be induced by ER stress. Further studies are needed to investigate whether ATF4 requires PTMs to activate its function and how this process occurs in *A/A* cells under ER stress conditions. Third, similar to previously reported results (Han et al., 2013), we observed weak but distinct induction of apoptotic cell death through Annexin V and 7-AAD analysis in ATF4-overexpressing *A/A* cells under normal conditions (Le et al., submitted to *Molecules and Cells*). Therefore, transient ATF4 OE might negatively impact mitochondrial functions. Further research is necessary to explore whether the toxic effects of ATF4 OE can counteract its beneficial effects and to understand the underlying mechanisms in *A/A* cells under normal conditions. However, this report focused on the roles of ATF4 in *A/A* cells under ER stress conditions. Therefore, all the studies described above are not necessary to draw the conclusions of this report.

PGC1α upregulates NRF1 and 2α, which promote transcription of nuclear DNA-encoded OXPHOS complex genes (such as those encoding all complex I subunits, except for mt-ND1-6) (Chen et al., 2009, Scarpulla et al., 2012). In addition to TFAM, the PGC1α-NRF1/2α pathway also upregulates the mitochondrial TFs B1 (TFB1M) and B2 (TFB2M), which are essential components of the mtDNA transcriptional machinery (Chen et al., 2009, Scarpulla et al., 2012). Therefore, levels of TFAM, TFB1M, and TFB2M are related to the expression of mtDNA-encoded OXPHOS complex genes (such as those encoding mt-ND1-6 in complex I) (Chen et al., 2009, Scarpulla et al., 2012). Based on the results presented in Figures 1-4 and S1-S3, and from previous reports described above, we speculate that impairment of downstream transcriptional cascades (such as the Nrf2-PGC1α-NRF1/2α-TFAM/TFB1M/TFB2M pathway) in addition to the eIF2α phosphorylation-ATF4 pathway downregulates OXPHOS complex proteins and thereby dysregulates mitochondrial functions in *A/A* cells during ER stress. However, we did not elucidate the detailed molecular mechanisms by which the OXPHOS complex I and II subunits and cytochrome C, but no other complex subunits, are downregulated or determine whether expression of TFB1M and TFB2M is changed in *A/A* cells during ER stress. These issues require further investigation.

Previous reports (Hu et al., 2018, Iurlaro and Munoz-Pinedo, 2016, Quiros et al., 2017) and our results indicate that ATF4 plays a dual role in mitochondrial homeostasis, acting as both a protective factor and a potential trigger for apoptosis during ER stress. Prolonged ER stress can lead to sustained ATF4 expression, which may result in mitochondrial impairments, such as decreased MMP (Fig. 4J) and ATP levels (Han et al., 2013). This mitochondrial dysfunction can trigger apoptotic pathways. However, it is believed that these events are induced by the cooperation of ATF4 and its downstream target, CHOP (Hu et al., 2018, Iurlaro and Munoz-Pinedo, 2016). ATF4 and CHOP activate apoptotic pathways by inhibiting BCL-2, BCL-XL, and MCL-1, while upregulating BIM, NOXA, and PUMA, which regulate BAX-BAK-mediated mitochondrial outer membrane permeabilization. This leads to MMP depletion, cytochrome c release, and subsequent activation of the caspase cascade (Hu et al., 2018, Iurlaro and Munoz-Pinedo, 2016).

Under ER stress, however, ATF4 also activates the expression of genes involved in serine/glycine biosynthesis, the 1-carbon metabolic pathway, purine synthesis, and GSH- and NADPH-mediated oxidative stress protection, all of which are essential for maintaining mitochondrial homeostasis (Ben-Sahra et al., 2016, DeNicola et al., 2015, Harding et al., 2003, Kasai et al., 2019, Wang et al., 2022). Similarly, we report that the eIF2α phosphorylation-ATF4-Nrf2 axis induces the transcriptional expression of genes involved in GSH synthesis and the mitochondrial 1-carbon metabolic pathway supplying NADPH (Le et al., submitted to *Molecules and Cells*). Additionally, ATF4 regulates mitochondrial function by inducing mitochondrial chaperones such as LONP1, HSPA9, and HSPD1 (Jiang et al., 2020). Furthermore, we demonstrate that ATF4 is required for the expression of multiple TFs, including Nrf2, and their target genes responsible for mitochondrial dynamics, mtDNA replication, and OXPHOS complex during ER stress. However, increased stress duration and severity can shift ATF4's role toward promoting apoptosis with CHOP induction (Rutkowski et al., 2006). Conversely, the interaction of ATF4 with Nrf2, which represses CHOP expression (Zong et al., 2012), or ATF4-dependent Nrf2 expression (Sarcinelli et al., 2020), as demonstrated in our results, can prevent or mitigate CHOP-driven apoptosis (Kasai et al., 2019). However, further studies are needed to determine which cellular states allow ATF4 to function as a protective factor in maintaining mitochondrial homeostasis during ER stress. Thus, the balance between ATF4's protective and proapoptotic functions can be influenced by context-specific factors related to cellular status.

In conclusion, we found that eIF2α phosphorylation suppresses the impairment of mitochondrial homeostasis upon ER stress via transcriptional reprogramming induced by its downstream TFs. Therefore, targeting eIF2α phosphorylation may be a potential approach to treat rapidly growing tumors, which rely on UPR activation and mitochondrial functions to adapt to stressful microenvironments (Chen and Cubillos-Ruiz, 2021, Wang and Kaufman, 2014).

## Funding and Support

This work was funded by the Basic Science Research Program (2020R1F1A1066088, 2022R1A2C1010449, and RS-2024-00408826 to S.H.B.) of the National Research Foundation of Korea, which is funded by the Korean government.

## AUTHOR CONTRIBUTIONS

**Sung Hoon Back:** Writing—review and editing, writing—original draft, supervision, resources, methodology, investigation, funding acquisition, data curation, and conceptualization. **Jeong Kon Seo:** Writing—review and editing, writing—original draft, software, resources, methodology, investigation, formal analysis, and data curation. **Hien Thi Le:** Writing—review and editing, writing—original draft, visualization, validation, methodology, investigation, formal analysis, data curation, and conceptualization. **Kyunggon Kim:** Writing—review and editing, writing—original draft, visualization, validation, software, resources, methodology, investigation, formal analysis, and data curation. **Jiyoung Yu:** Writing—original draft, visualization, methodology, formal analysis, and data curation. **Hee Sung Ahn:** Writing—original draft, visualization, validation, and data curation. **Mi-Jeong Kim:** Visualization, validation, resources, and data curation. **In Gyeong Chae:** Visualization, validation, investigation, and data curation. **Hyun-Nam Cho:** Visualization, methodology, investigation, data curation, and conceptualization. **Juhee Kim:** Visualization, validation, software, and data curation. **Hye-Kyung Park:** Resources, methodology. **Hyuk Nam Kwon:** Visualization, software, resources, methodology, and data curation. **Han-Jung Chae:** Resources, methodology. **Byoung Heon Kang:** Resources, methodology.

## DECLARATION OF COMPETING INTERESTS

The authors declare that they have no known competing financial interests or personal relationships that could have appeared to influence the work reported in this paper.

## ORCID

Hien Thi Le: https://orcid.org/0000-0002-7638-4798

Jiyoung Yu: https://orcid.org/0000-0002-3953-200X

Hee Sung Ahn: https://orcid.org/0000-0003-2241-6536

Mi-Jeong Kim: https://orcid.org/0000-0003-1610-7649

In Gyeong Chae: https://orcid.org/0000-0002-4496-4448

Hyun-Nam Cho: https://orcid.org/0009-0003-2099-214X

Juhee Kim: https://orcid.org/0009-0005-5400-0238

Hye-Kyung Park: https://orcid.org/0000-0001-6583-2623

Hyuk Nam Kwon: https://orcid.org/0000-0003-4096-4712

Han-Jung Chae: https://orcid.org/0000-0003-4190-9889

Byoung Heon Kang: https://orcid.org/0000-0001-5902-0549

Jeong Kon Seo: https://orcid.org/0000-0001-6165-2633

Kyunggon Kim: https://orcid.org/0000-0002-3072-5331

Sung Hoon Back: https://orcid.org/0000-0001-8029-1298

## Footnotes

Appendix A Supplementary data associated with this article can be found in the online version at doi:10.1016/j.mocell.2024.100176.
