# Supplementary material for: eIF2α phosphorylation-ATF4 axis-mediated transcriptional reprogramming mitigates mitochondrial impairment during ER stress
Source: Mol Cells. 2025 Jan 3;48(2):100176. doi: 10.1016/j.mocell.2024.100176 (PMC11786836; doi:10.1016/j.mocell.2024.100176)
Supplement: Supplementary file 1 — Supplementary material [file mmc1.pdf]

Supplementary figures and their legends

eIF2α Phosphorylation-ATF4 Axis-Mediated Transcriptional Reprogramming Mitigates Mitochondrial Impairment During ER Stress

Hien Thi Le[a,*], Jiyoung Yu[b,*], Hee Sung Ahn[c], Mi-Jeong Kim[a], In Gyeong Chae[a], Hyun-Nam Cho[a], Juhee Kim[a], Hye-Kyung Park[d], Hyuk Nam Kwon[a], Han-Jung Chae[e], Byoung Heon Kang[d], Jeong Kon Seo[f, #], Kyunggon Kim[g, ##], Sung Hoon Back[a, h, ###]

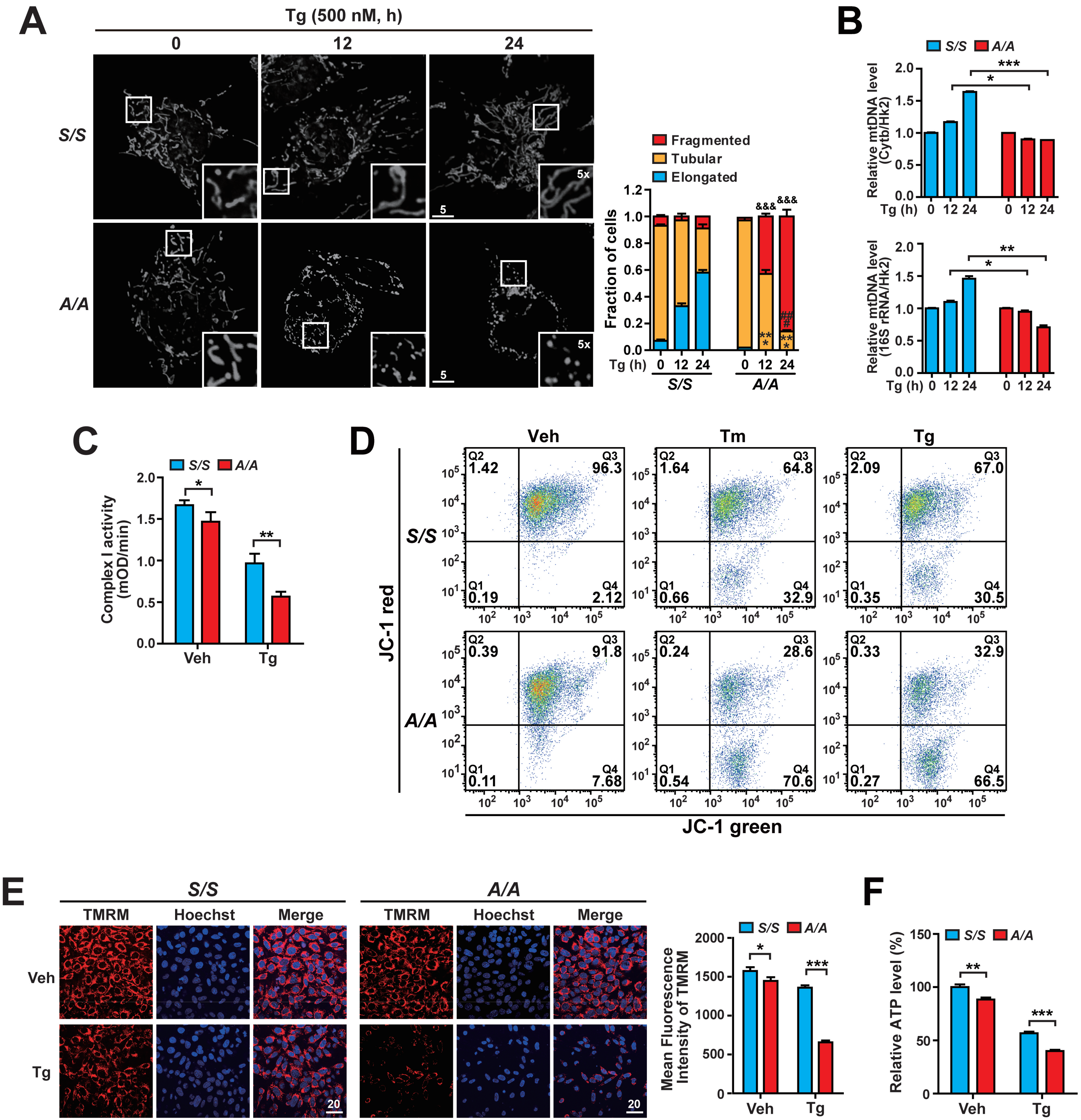
A
Tg (500 nM, h)
0
12
24
S/S
A/A
5x
5
Fragmented
Tubular
Elongated
Fraction of cells
Tg (h)
S/S
A/A
&&&
B
S/S
A/A
Relative mtDNA level (Cytb/Hk2)
Relative mtDNA level (16S rRNA/Hk2)
Tg (h)
C
Complex I activity (mOD/min)
Veh
Tg
D
Veh
Tm
Tg
JC-1 red
JC-1 green
Q1
Q2
Q3
Q4
1.42
96.3
0.19
2.12
1.64
64.8
0.66
32.9
2.09
67.0
0.35
30.5
0.39
91.8
0.11
7.68
0.24
28.6
0.54
70.6
0.33
32.9
0.27
66.5
E
TMRM
Hoechst
Merge
20
Mean Fluorescence Intensity of TMRM
F
Relative ATP level (%)

Fig. S1. eIF2α phosphorylation is required for mitochondrial homeostasis in Tg-treated *A/A* MEFs. (A) Representative MitoTracker Red staining images of *S/S* and *A/A* MEFs. Cells were treated with Veh (DMSO) or Tg (500 nM) for the indicated durations and stained with MitoTracker Red (white) for the last 30 min. The inset shows a 5× magnified image of the white boxed area. Scale bar: 5 μm. The graph shows the fractions of cells containing fragmented, tubular, and elongated mitochondria among *S/S* and *A/A* MEFs treated with Tg. Data are presented as mean ± SEM (at least 50 cells per condition). ***p < 0.001, *S/S* vs. *A/A* at each time point of "Elongated"; $^{###}$p < 0.001, *S/S* vs. *A/A* at each time point of "Tubular"; $^{\&\&\&}$p < 0.001, *S/S* vs. *A/A* at each time point of "Fragmented". **(B)** qPCR analysis of mtDNA levels in *S/S* and *A/A* MEFs treated with Tg for the indicated durations. The mtDNA (*mt-cytb* and *mt-16s rRNA*) levels were normalized by the nuclear DNA (*Hk2*) level in each sample. Data are presented as mean ± SEM (n = 3). *p < 0.05, **p < 0.01, and ***p < 0.001, *S/S* vs. *A/A* at each time point. **(C)** Mitochondrial complex I activity in *S/S* and *A/A* MEFs treated with Veh or Tg for 24 h. Data are presented as mean ± SEM (n = 3). *p < 0.05 and **p < 0.01, *S/S* vs. *A/A*. **(D)** Representative flow cytometric analysis images of JC-1-stained *S/S* and *A/A* MEFs treated with Veh, Tm, or Tg for 24 h. The values shown represent the means (%) (n = 3). **(E)** Representative TMRM staining images of *S/S* and *A/A* MEFs. Cells were treated with Veh or Tg for 24 h and stained with TMRM (red) and Hoechst 33258 (blue) for the last 30 min. Scale bar: 20 μm. The graph depicts quantification of the MFI of TMRM. Data are presented as mean ± SEM (n = 3, 15 random fields per condition). *p < 0.05 and ***p < 0.001, *S/S* vs. *A/A*. **(F)** Measurement of total ATP levels in *S/S* and *A/A* MEFs treated with Veh or Tg for 24

h. Data are presented as mean ± SEM (n = 3). $^{**}p < 0.01$ and $^{***}p < 0.001$, *S/S* vs. *A/A*.

Supple Fig-02

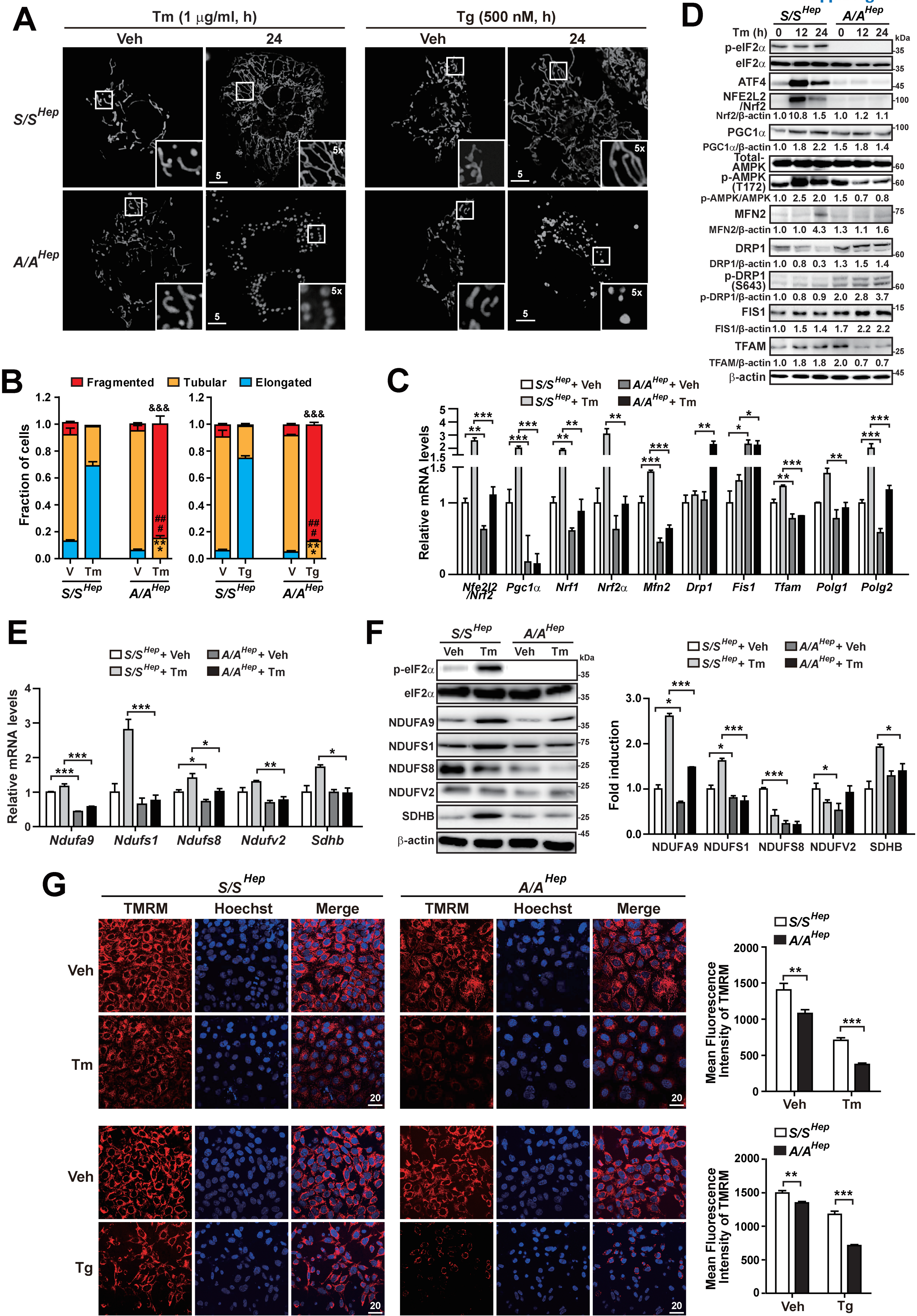

Fig. S2. eIF2α phosphorylation is required for proper expression of genes responsible for mitochondrial homeostasis in immortalized hepatocytes during ER stress. (A) Representative MitoTracker Red staining images of S/S^{Hep} and A/A^{Hep} cells. Cells were treated with Veh (DMSO), Tm (1 μg/mL), or Tg (500 nM) for 24 h and stained with MitoTracker Red (white) for the last 30 min. The inset shows a 5× magnified image of the white boxed area. Scale bar: 5 μm. **(B)** The graph shows the fractions of cells containing fragmented, tubular, and elongated mitochondria among S/S^{Hep} and A/A^{Hep} cells treated with Tm or Tg. Data are presented as mean ± SEM (at least 50 cells per condition). ***p < 0.001, S/S^{Hep} vs. A/A^{Hep} in "Elongated"; $^{###}$p < 0.001, S/S^{Hep} vs. A/A^{Hep} in "Tubular"; $^{\&\&\&}$p < 0.001, S/S^{Hep} vs. A/A^{Hep} in "Fragmented". **(C)** Quantitative RT-PCR analysis of mRNA levels of mitochondrial dynamics- and mtDNA replication-related genes in S/S^{Hep} and A/A^{Hep} cells treated with Veh or Tm for 24 h. Data are presented as mean ± SEM (n = 3). **p < 0.01 and ***p < 0.001, S/S^{Hep} vs. A/A^{Hep}. **(D)** WB analysis of mitochondrial dynamics- and mtDNA replication-related proteins in lysates of S/S^{Hep} and A/A^{Hep} cells treated with Tm for the indicated durations. Protein levels normalized by β-act or AMPK levels are shown below the panels. **(E)** Quantitative RT-PCR analysis of mRNA expression of electron transport chain/OXPHOS complex I genes in S/S^{Hep} and A/A^{Hep} cells treated with Vec or Tm for 24 h. Data are presented as mean ± SEM (n = 3). *p < 0.05, **p < 0.01, and ***p < 0.001, S/S^{Hep} vs. A/A^{Hep}. **(F)** WB analysis of OXPHOS complex I subunits in lysates of S/S^{Hep} and A/A^{Hep} cells treated with Veh or Tm for 24 h. The graph depicts the protein level normalized by the β-act level (n = 3). *p < 0.05 and ***p < 0.001, S/S^{Hep} vs. A/A^{Hep}. **(G)** Representative TMRM staining images of S/S^{Hep} and A/A^{Hep} cells. Cells were treated with Veh, Tm, or Tg for 24 h and stained with TMRM (red) and Hoechst 33258

(blue) for the last 30 min. Scale bar: 20 μm. The graph depicts quantification of the MFI of TMRM. Data are presented as mean ± SEM (n = 3, 15 random fields per condition). $^{**}p < 0.01$ and $^{***}p < 0.001$, S/S^{Hep} vs. A/A^{Hep}.

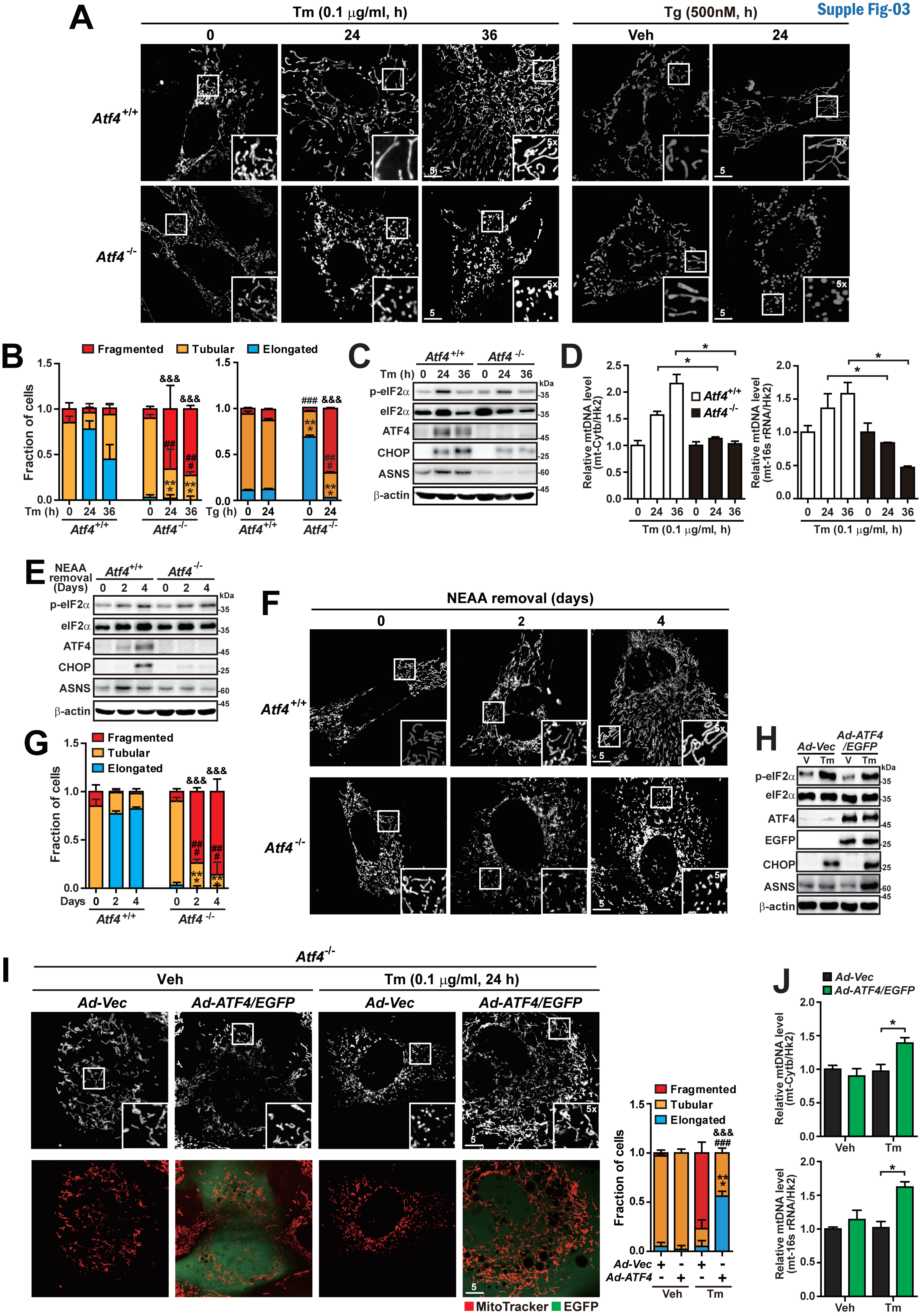
Supple Fig-03
A
Tm (0.1 μg/ml, h)
Tg (500nM, h)
0
24
36
Veh
24
Atf4+/+
Atf4-/-
5x
5
B
Fragmented
Tubular
Elongated
Fraction of cells
Tm (h)
Tg (h)
&&&
##
###
C
Atf4+/+
Atf4-/-
Tm (h)
p-eIF2α
eIF2α
ATF4
CHOP
ASNS
β-actin
kDa
D
Relative mtDNA level (mt-Cytb/Hk2)
Relative mtDNA level (mt-16s rRNA/Hk2)
Tm (0.1 μg/ml, h)
E
NEAA removal (Days)
F
NEAA removal (days)
G
Days
H
Ad-Vec
Ad-ATF4/EGFP
V
Tm
EGFP
I
Veh
Tm (0.1 μg/ml, 24 h)
MitoTracker
J
Ad-ATF4

Fig. S3. ATF4 is responsible for mitochondrial dynamics and mtDNA replication during ER stress or NEAA deprivation. (A) Representative MitoTracker Red staining images of *ATF4*$^{+/+}$ and *ATF4*$^{-/-}$ MEFs. Cells were treated with Tm (0.1 μg/mL) or Tg (500 nM) for the indicated durations and stained with MitoTracker Red (white) for the last 30 min. The inset shows a 5× magnified image of the white boxed area. Scale bar: 5 μm. **(B)** The graph shows the fractions of cells containing fragmented, tubular, and elongated mitochondria among *ATF4*$^{+/+}$ and *ATF4*$^{-/-}$ MEFs treated with Tm. Data are presented as mean ± SEM (at least 50 cells per condition). ***$p < 0.001$, *ATF4*$^{+/+}$ vs. *ATF4*$^{-/-}$ at each time point of “Elongated”; $^{\#\#}p < 0.01$ and $^{\#\#\#}p < 0.001$, *ATF4*$^{+/+}$ vs. *ATF4*$^{-/-}$ at each time point of “Tubular”; $^{\&\&\&}p < 0.001$, *ATF4*$^{+/+}$ vs. *ATF4*$^{-/-}$ at each time point of “Fragmented”. **(C)** WB analysis of p-eIF2α, eIF2α, and downstream proteins in lysates of *ATF4*$^{+/+}$ and *ATF4*$^{-/-}$ MEFs treated with Tm (0.1 μg/mL) for the indicated durations. **(D)** qPCR analysis of mtDNA levels in *ATF4*$^{+/+}$ and *ATF4*$^{-/-}$ MEFs treated with Tm for the indicated durations. The mtDNA (*mt-cytb* and *mt-16s rRNA*) levels were normalized by the nuclear DNA (*Hk2*) level in each sample. Data are presented as mean ± SEM ($n = 3$). *$p < 0.05$, *ATF4*$^{+/+}$ vs. *ATF4*$^{-/-}$ at each time point. **(E)** WB analysis of lysates of *ATF4*$^{+/+}$ and *ATF4*$^{-/-}$ MEFs cultivated without addition of NEAAs for the indicated durations. **(F)** Representative MitoTracker Red staining images of *ATF4*$^{+/+}$ and *ATF4*$^{-/-}$ MEFs. Cells were cultivated in DMEM without addition of NEAAs for the indicated durations and stained with MitoTracker Red (white) for the last 30 min. The inset shows a 5× magnified image of the white boxed area. Scale bar: 5 μm. **(G)** The graph shows the fractions of cells containing fragmented, tubular, and elongated mitochondria among *ATF4*$^{+/+}$ and *ATF4*$^{-/-}$ MEFs. Data are presented as mean ± SEM (at least 50 cells per condition). ***$p < 0.001$, *ATF4*$^{+/+}$ vs.

ATF4$^{-/-}$ at each time point of “Elongated”; $^{###}p < 0.001$, *ATF4*$^{+/+}$ vs. *ATF4*$^{-/-}$ at each time point of “Tubular”; $^{\&\&\&}p < 0.001$, *ATF4*$^{+/+}$ vs. *ATF4*$^{-/-}$ at each time point of “Fragmented”. **(H)** WB analysis of lysates of Vec- or ATF4/EGFP-overexpressing *ATF4*$^{-/-}$ MEFs treated with or without Tm for 24 h. **(I)** Representative MitoTracker Red staining images of Vec- or ATF4/EGFP-overexpressing *ATF4*$^{-/-}$ MEFs. Cells were treated with Veh or Tm (0.1 μg/mL) for 24 h and stained with MitoTracker Red (white) for the last 30 min. Expression of ATF4 is indicated by the green fluorescence of EGFP. The inset shows a 5× magnified image of the white boxed area. Scale bar: 5 μm. The graph shows the fractions of cells containing fragmented, tubular, and elongated mitochondria among Vec- or ATF4/EGFP-overexpressing *A/A* MEFs treated with Veh or Tm. Data are presented as mean ± SEM (at least 50 cells per condition). $^{***}p < 0.001$, *Vec* vs. *ATF4/EGFP* in “Elongated”; $^{###}p < 0.001$, *Vec* vs. *ATF4/EGFP* in “Tubular”; $^{\&\&\&}p < 0.001$, *Vec* vs. *ATF4/EGFP* in “Fragmented”. **(J)** qPCR analysis of mtDNA levels in Vec- or ATF4/EGFP-overexpressing *A/A* MEFs treated with Tm for 24 h. The mtDNA (*mt-cytb* and *mt-16s rRNA*) levels were normalized by the nuclear DNA (*Hk2*) level in each sample. Data are presented as mean ± SEM (n = 3). $^{*}p < 0.05$, *Vec* vs. *ATF4/EGFP*.

Supple Fig-4

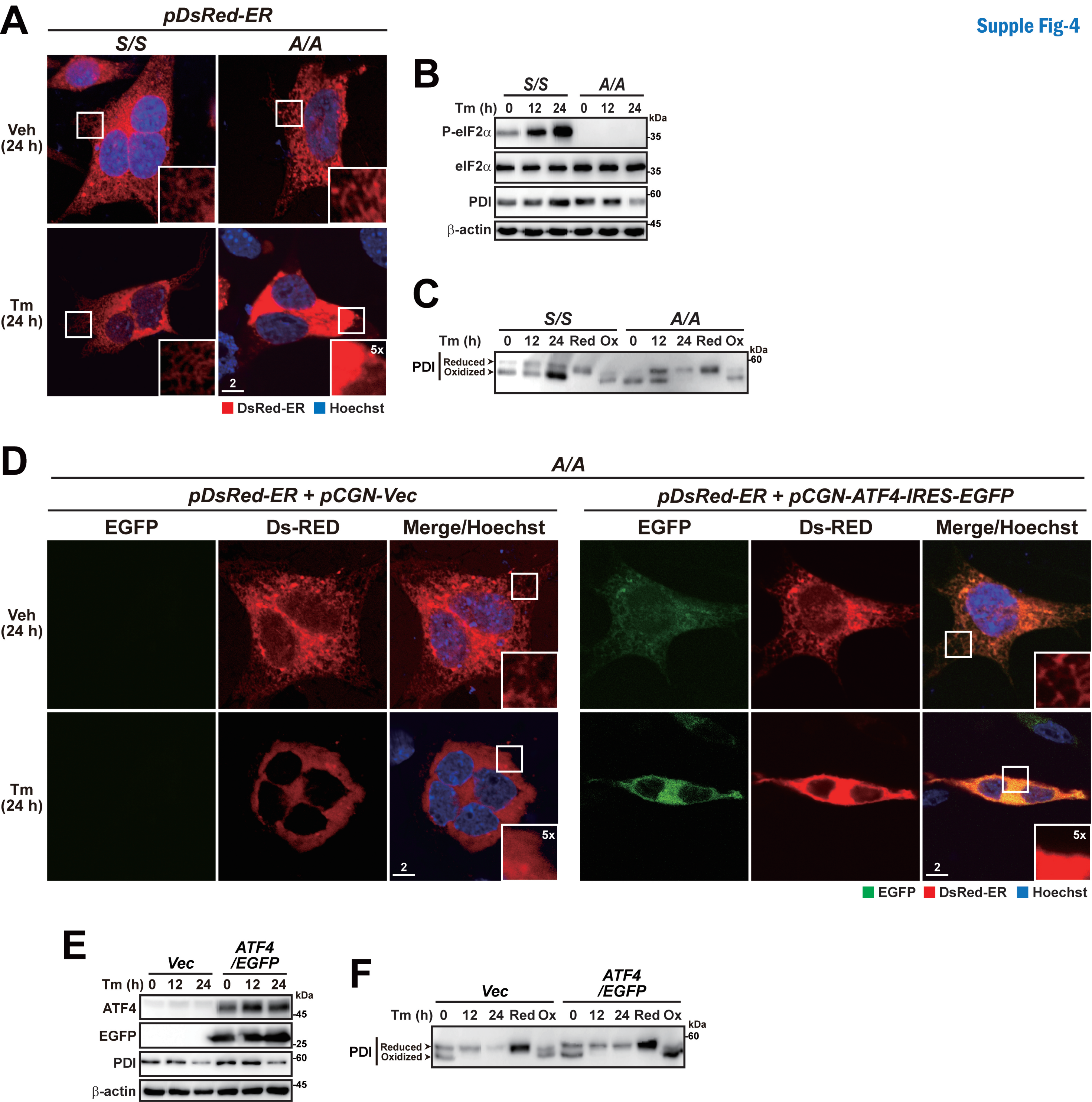
A
pDsRed-ER
S/S
A/A
Veh (24 h)
Tm (24 h)
5x
2
DsRed-ER
Hoechst
B
S/S
A/A
Tm (h) 0 12 24 0 12 24
kDa
P-eIF2α
eIF2α
PDI
β-actin
35
35
60
45
C
S/S
A/A
Tm (h) 0 12 24 Red Ox 0 12 24 Red Ox
kDa
60
PDI
Reduced
Oxidized
D
A/A
pDsRed-ER + pCGN-Vec
pDsRed-ER + pCGN-ATF4-IRES-EGFP
EGFP
Ds-RED
Merge/Hoechst
EGFP
Ds-RED
Merge/Hoechst
Veh (24 h)
Tm (24 h)
5x
2
5x
2
EGFP
DsRed-ER
Hoechst
E
Vec
ATF4 /EGFP
Tm (h) 0 12 24 0 12 24
kDa
ATF4
EGFP
PDI
β-actin
45
25
60
45
F
Vec
ATF4 /EGFP
Tm (h) 0 12 24 Red Ox 0 12 24 Red Ox
kDa
60
PDI
Reduced
Oxidized

Fig. S4. ATF4 OE does not prevent formation of an aberrant ER structure or impairment of ER function in *A/A* cells during ER stress. (A) Representative microscopic images of the ER in DsRed-ER-expressing *S/S* and *A/A* MEFs. Cells transfected with a DsRed-ER-expressing plasmid (*pDsRed-ER*) for 24 h were treated with Veh or Tm for 24 h and stained with Hoechst 33258 (blue) for the last 30 min. ER structures were visualized by red fluorescence. The inset shows a 5× magnified image of the white boxed area. Scale bar: 2 μm. **(B)** WB analysis of the ER chaperone PDI in lysates of *S/S* and *A/A* MEFs treated with Tm for the indicated durations. **(C)** Analysis of the redox status of PDI in *S/S* and *A/A* MEFs. Cells were treated with Tm for the indicated durations. Protein lysates were then prepared as described in the Materials and methods. Formation of disulfides was blocked by alkylating free thiols with AMS. For reduced (Red) and oxidized (Ox) controls, lysates were incubated with DDT and diamide, respectively. Samples were electrophoresed on a non-reducing SDS-PAGE gel and subjected to WB analysis. **(D)** Representative microscopic images of the ER in Vec- or ATF4/EGFP-overexpressing *A/A* MEFs. *A/A* MEFs co-transfected with *pDsRed-ER* plus *pCGN-Vec* or *pDsRed-ER* plus *pCGN-ATF4-IRES-EGFP* for 24 h were treated with Veh or Tm for 24 h and stained with Hoechst 33258 (blue) for the last 30 min. Expression of ATF4 is indicated by the green fluorescence of EGFP. ER structures were visualized by red fluorescence. The inset shows a 5× magnified image of the white boxed area. Scale bar: 2 μm. **(E)** WB analysis of ATF4, EGFP, and PDI in lysates of Vec- or ATF4/EGFP-overexpressing *A/A* MEFs. *A/A* MEFs were transfected with *pCGN-Vec* or *pCGN-ATF4-IRES-EGFP* for 24 h and treated with Tm for the indicated durations. Cell lysates were prepared and subjected to WB analysis of the indicated proteins. **(F)** Analysis of the redox status of PDI in Vec- or ATF4/EGFP-

overexpressing *A/A* MEFs. Cells were treated with Tm for the indicated durations. Protein lysates were then prepared as described in the Materials and methods. Samples were electrophoresed on a non-reducing SDS-PAGE gel and subjected to WB analysis.
